# Supplementary material for: Real-time monitoring polymerization degree of organic photovoltaic materials toward no batch-to-batch variations in device performance
Source: Nat Commun. 2024 Feb 10;15:1248. doi: 10.1038/s41467-024-45510-w (PMC10858892; doi:10.1038/s41467-024-45510-w)
Supplement: Supplementary file 1 — Supplementary Information [file 41467_2024_45510_MOESM1_ESM.pdf]

Supplementary Information

Real-time monitoring polymerization degree of organic photovoltaic materials toward no batch-to-batch variations in device performance

Lin-Yong Xu[1 †], Wei Wang[1 †], Xinrong Yang[1], Shanshan Wang[1], Yiming Shao[1], Mingxia Chen[1], Rui Sun[1*], Jie Min[1*]

[1] The Institute for Advanced Studies, Wuhan University, Wuhan 430072, China

† These authors contributed equally.

E-mail: sun.rui@whu.edu.cn, min.jie@whu.edu.cn

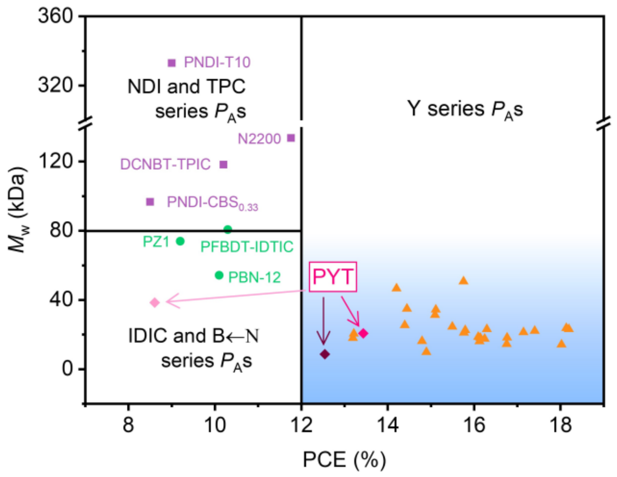

Supplementary Fig. 1. Weight-average molecular weight *versus* PCE in the previously reported all-polymer systems based on various polymer acceptors.

Supplementary Table 1. The device efficiencies and molecular mass parameters of the previously reported Y-series polymer acceptors.

Material	PCE (%)	M_n (kDa)	M_w (kDa)	$Đ$	Ref.
PTz-BO	15.76	16.7	50.5	3.0	1
PYT-82	18.03	8.9	14.0	1.57	1
PY-1S1Se	18.2	12.7	22.9	1.8	2
PY-SSe-V	18.14	10.5	23.5	2.23	3
PYT-EH20	14.8	10.2	16.0	1.35	4
PY-82	17.15	10.7	21.1	1.57	5
PJTVT	16.13	10.2	15.9	1.56	6
PYT-1S1Se	16.3	12.7	22.9	1.80	7
PYT-2Se	15.5	12.9	24.3	1.88	7
PZT-γ	15.8	10.6	22.3	2.1	8
PY-DT	16.76	7.2	14.3	1.98	5
PYF-IT	15.1	22.2	31.1	1.4	9
PBTIC-γ-TSe	15.77	11.6	20.9	1.97	10
PT-YTz	16.15	10.0	17.9	1.78	11
PYDT-2F	16.25	10.9	17.3	1.59	12
PYDT-3F	17.41	12.1	21.9	1.81	9
PYDT-4F	16.77	11.4	18.0	1.58	9
PY3Se-1V	13.2	9.5	17.8	1.87	13
PYSe-TCl20	14.21	22.1	46.4	2.1	14
PTz-C1	14.9	5.1	9.6	1.9	15
RRg-C20	15.12	19.0	34.2	1.8	16
PY2Se-Cl	16.1	10.5	18.4	1.75	13
PBN25	14.4	11.2	25.2	2.25	17
PYN-BDTF	13.22	13.5	20.3	1.50	18
PF5-Y5	14.45	16.5	34.7	2.10	19

PYT_L	12.55	7.2	8.4	1.16	20
PYT_M	13.44	12.3	20.5	1.67	20
PYT_H	8.61	20.6	38.3	1.86	20
DCNBT-TPIC	10.22	43.8	118.2	2.7	19
PNDI-$CBS_{0.33}$	8.5	38.7	96.8	2.5	21
N2200	11.76	75.1	133.7	1.78	22
PZ1	9.2	33.7	73.8	2.19	23
PNDI-T10	9.0	66.6	333.0	5.0	23
PBN-12	10.1	37.8	54.1	1.43	24
PFBDT-IDTIC	10.3	35.2	80.5	2.3	25

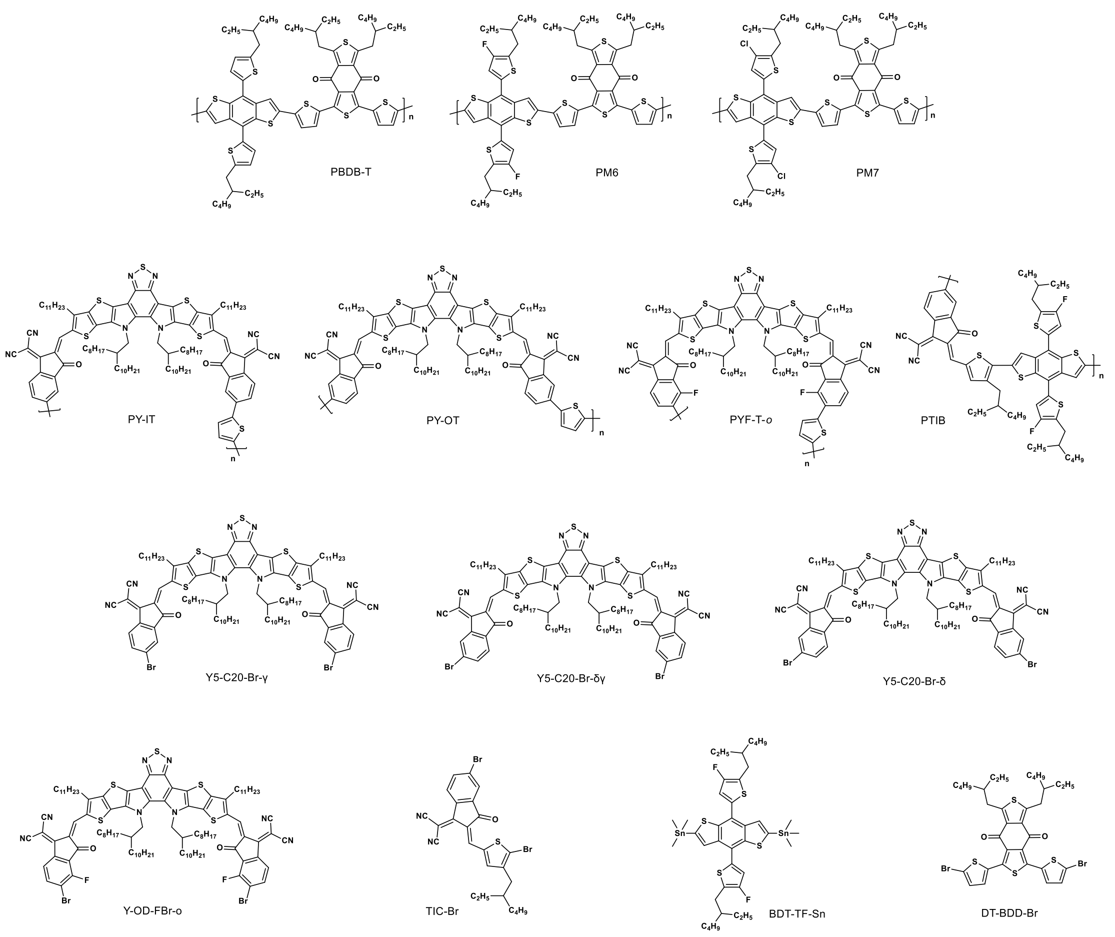

Supplementary Fig. 2. Chemical structures of the polymer donors PBDB-T, PM6, PM7, polymer acceptors PY-IT, PY-OT, PYF-T-*o*, PTIB, Y5-C20-Br isomer Y5-C20-Br-γ, Y5-C20-Br-δγ, Y5-C20-Br-δ, and monomers Y-OD-FBr-*o*, TIC-Br, BDT-TF-Sn, DT-BDD-Br.

Supplementary Table 2. The molecular mass parameters of the polymer donors PBDB-T, PM6 and PM7 and the PYT batches.

	PYT_L	PYT_M	PYT_H	PBDB-T	PM6	PM7
M_n (kDa)	8.1	6.5	8.9	52.2	45.4	39.4
M_w (kDa)	9.7	10.6	16.1	97.6	97.7	93.3
Đ	1.2	1.6	1.8	1.87	2.15	2.37

Supplementary Table 3. Summary of relevant photovoltaic parameters of the three all-polymer systems composed of the polymer donors (PBDB-T, PM6, and PM7) and the PYT batches, measured under one sun illumination.

Polymer donor	Sample	V_{OC} (V)	J_{SC} (mA cm^{-2})	FF (%)	PCE (PCE[a]) (%)
PBDB-T	PYT_L	0.867	22.71	65.81	12.96 (12.79)
	PYT_M	0.853	23.50	66.99	13.43 (13.15)
	PYT_H	0.877	22.12	72.55	14.07 (13.87)
PM6	PYT_L	0.935	21.83	66.83	13.64 (13.40)
	PYT_M	0.947	20.02	68.13	12.92 (12.49)
	PYT_H	0.955	17.71	65.93	11.15 (10.86)
PM7	PYT_L	0.940	17.40	45.56	7.45 (7.17)
	PYT_M	0.963	14.47	57.40	8.00 (7.76)
	PYT_H	0.976	12.95	59.80	7.56 (7.33)

[a] The statistics were obtained from over six devices.

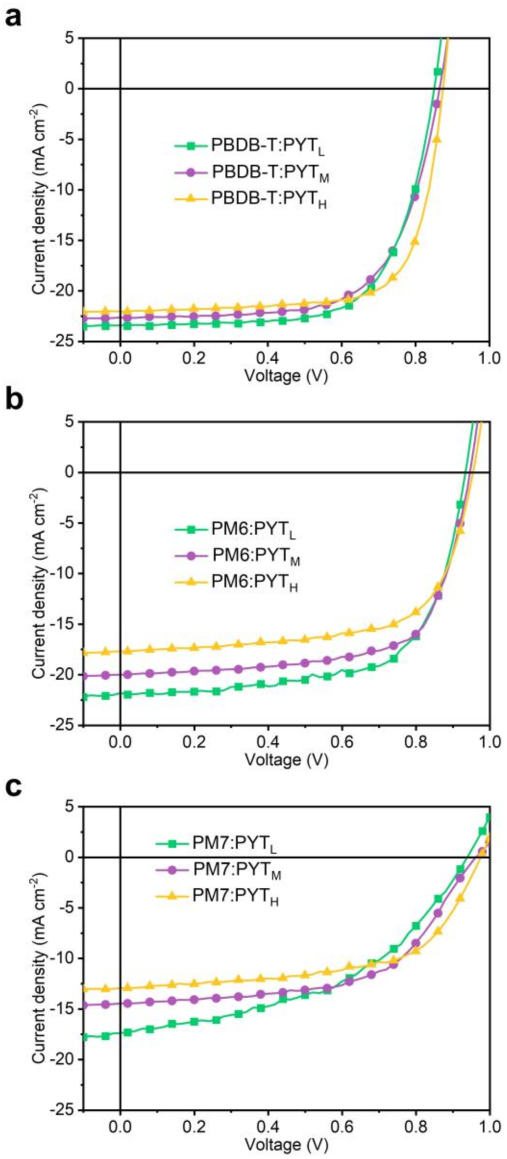

Supplementary Fig. 3. The relevant *J*-*V* curves of the three all-polymer systems composed of the polymer donors (**a** PBDB-T, **b** PM6, and **c** PM7) and the PYT batches, measured under one sun illumination.

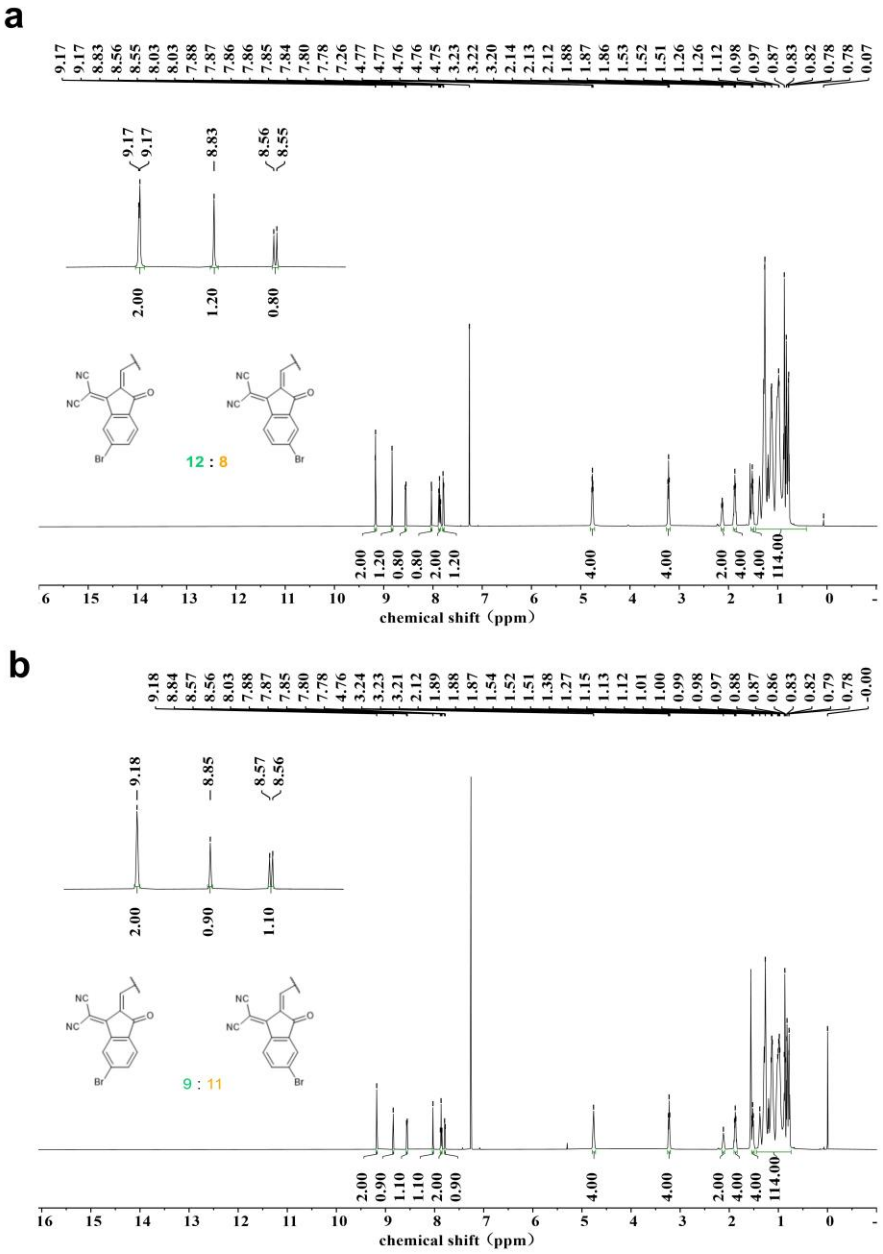

Supplementary Fig. 4. ^{1}H NMR spectrum of different monomer Y5-C20-Br batches (**a** for Batch δ and **b** for Batch γ, respectively).

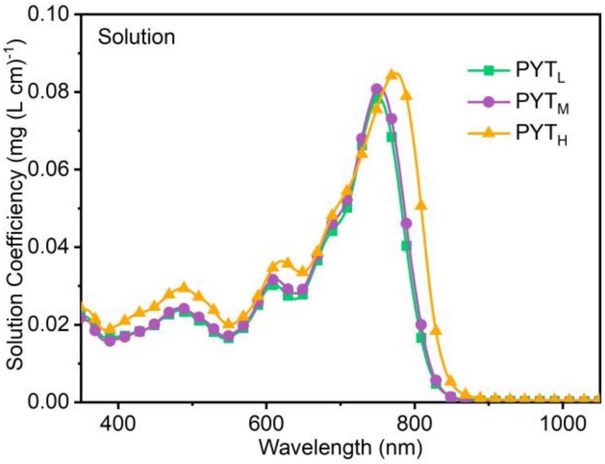

Supplementary Fig. 5. The measured absorption coefficients of the corresponding PYT batches in chloroform-diluted solutions with the same concentration (10^{-5} M).

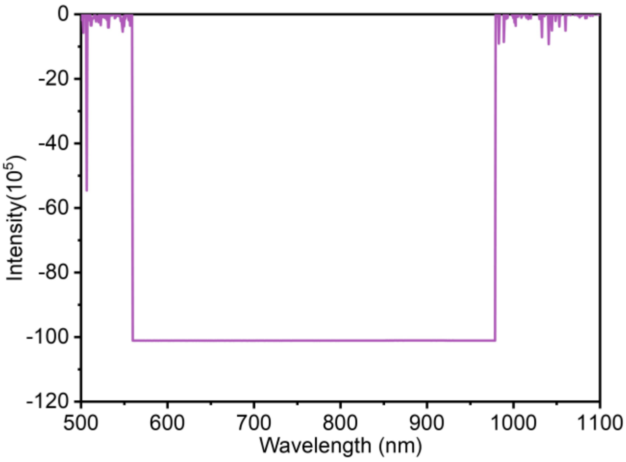

Supplementary Fig. 6. UV-vis absorption spectra of PYT polymerization system tested by an in-situ UV-vis setup. Note that absorbance beyond the range of instrumental measurements.

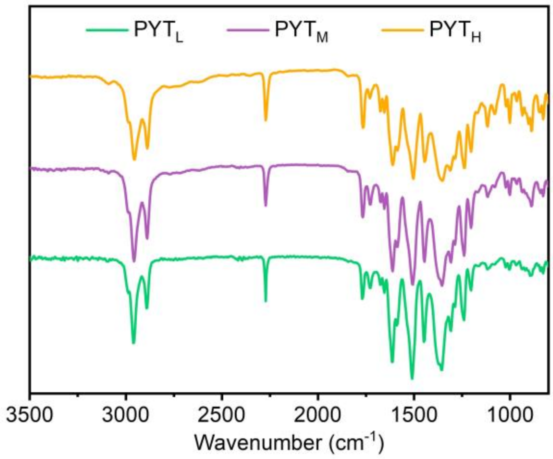

Supplementary Fig. 7. The IR spectra of the corresponding PYT batches in solutions.

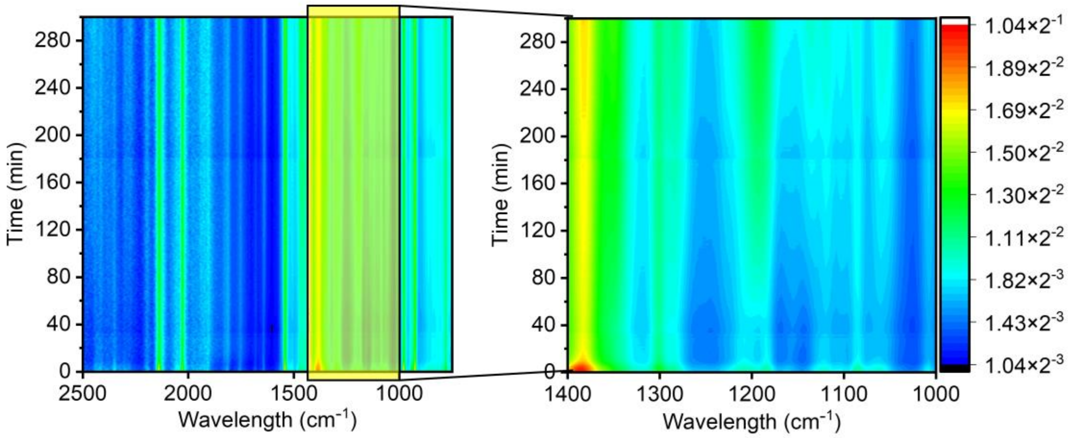

Supplementary Fig. 8. Time traces of the IR spectrum and relevant enlarged drawing tested by an in-situ IR setup. Note that the vibration intensity of the C-Br bond in the fingerprint region is weak and relevant spectrum is extremely complex.

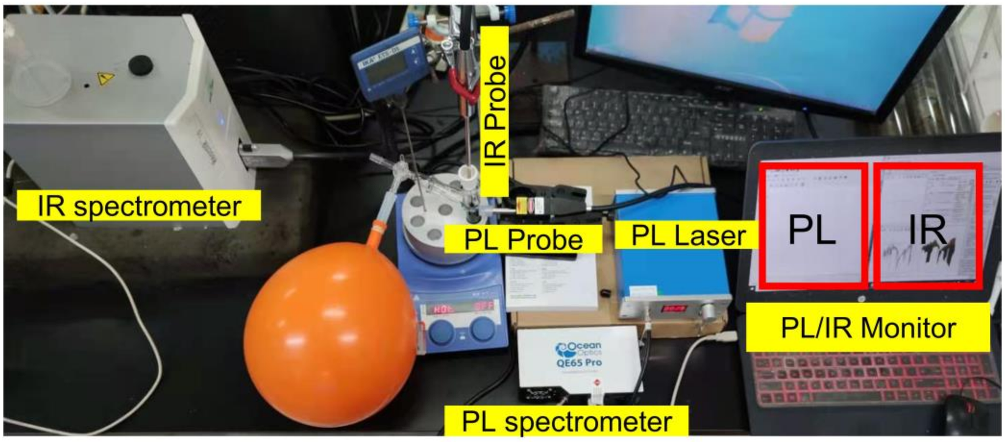

Supplementary Fig. 9. The experimental combined setup systems composed of in-situ IR and in-situ PL modules.

Supplementary Note 1.

The used analysis and processing programs (set operations in Python):

```
import os
import numpy as np
import winsound
import time
import matplotlib.pyplot as plt




def get_data(file_path, start=XXX, stop=XXX, div=XXX):
    with open(file_path, 'r', encoding='gbk') as f:
        data_all = f.readlines()[::-1]
        data = []
        for i in data_all:
            if '>>>>>Begin Spectral Data<<<<<' in i:
                break
            i = i.replace("\n", "")
            data.append(i.split("\t"))
        data = data[::-1]
    x = [float(i[0]) for i in data[start:stop]]
    y = [float(i[1]) for i in data[start:stop]]
    x1 = []
    m = x[0]
    while m <= x[-1]:
        x1.append(m)
        m += div
    return [x, x1, y]
```

```
def data_fitting(data, power=60, error=1, div=0.001, count=4):
    x1 = np.array(data[0])
    x2 = np.array(data[1])
    y = np.array(data[2])
    an = np.polyfit(x1, y, power)
    result = np.polyval(an, x2)
    y_max = np.max(result)
    h = float(y_max) / 2
    index = list(np.where(result == y_max))[0][0]
    x_max = float(x2[index])
    flag = True
    result1 = result[:index]
    result2 = result[index:]
    temp = error
    while flag:
        index1 = np.where((result1 >= h - temp) & (result1 <= h + temp))
        if len(list(index1)[0]) != 1:
            temp -= div
        else:
            flag = False
            pp1 = float(x2[list(index1)[0]])
    flag = True
    temp = error
    while flag:
        index1 = np.where((result2 >= h - temp) & (result2 <= h + temp))
        if len(list(index1)[0]) != 1:
            temp -= div
        else:
            flag = False
```

```
                pp2 = float(x2[list(index1 + index)[0]])
    return [round(x_max, count), round(y_max, count), round((pp1 + pp2) / 2,
count)]


last_data = []
t = 3
fig = plt.figure()
ax = fig.add_subplot(projection='3d')
ax.set(xlabel='PP', ylabel='PPC', zlabel='PI')
ax.set_xlim3d(xmin=XXX, xmax=XXX)
ax.set_ylim3d(ymin=XXX, ymax=XXX)
ax.set_zlim3d(zmin=XXXXX, zmax=XXXXX)
while True:
    try:
        a = os.listdir('data')
        time.sleep(t)
    except:
        time.sleep(t)
        a = os.listdir('data')
        time.sleep(t)
    for i in a:
        if i not in last_data:
            last_data.append(i)
            print(i)
            point = get_data('data//' + i)
            result = data_fitting(point)
            if XXX <= result[0] <= XXX and XXXXX <= result[1] <= XXXXX
and XXXXX <= result[2] <= XXXXX:
                print(i + ':' + str(result))
                ax.scatter(result[0], result[2], result[1], c='#ee0b0b', marker='*')
```

```
        winsound.PlaySound('sound.wav', flags=1)
        with open('Switch.txt', 'w', encoding='utf-8') as f:
            f.write('1')
else:
        with open('Switch.txt', 'r', encoding='utf-8') as f:
            play = int(f.read())
        if play == 1:
            winsound.PlaySound('sound.wav', flags=1)
        ax.scatter(result[0], result[2], result[1])
plt.pause(2)
```

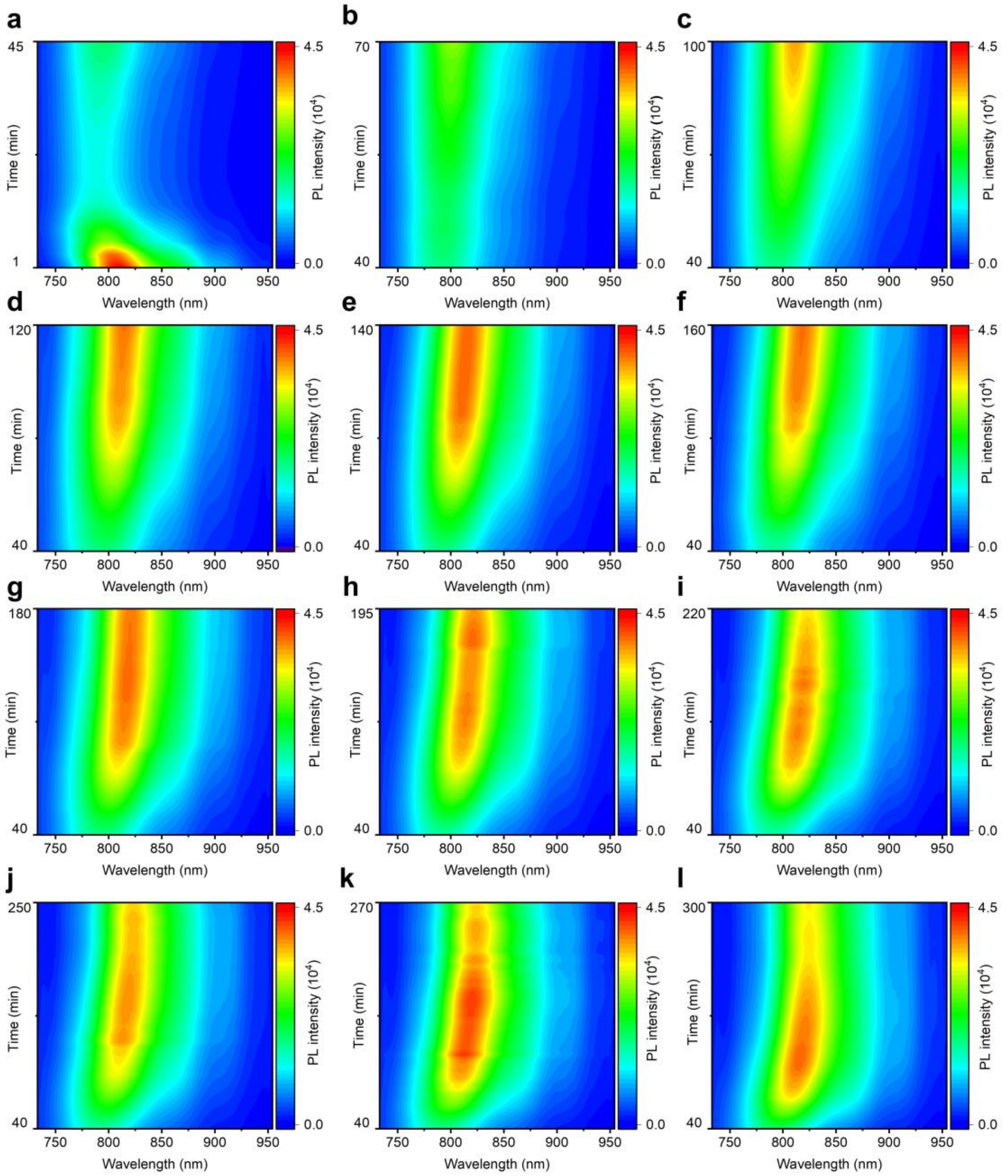

Supplementary Fig. 10. Two-dimensional PL spectrum maps of the PYT synthesis as a function of polymerization time, including **a** 45 mins, **b** 70 mins, **c** 100 mins, **d** 120 mins, **e** 140 mins, **f** 160 mins, **g** 180 mins, **h** 195 mins, **i** 220 mins, **j** 250 mins, **k** 270 mins, **l** 300 mins.

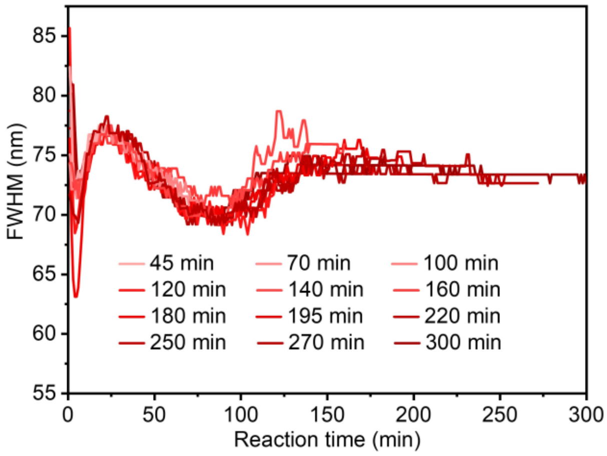

Supplementary Fig. 11. The FWHM curves extracted from the PL spectra tested by the in-situ PL system with different polymerization reaction times.

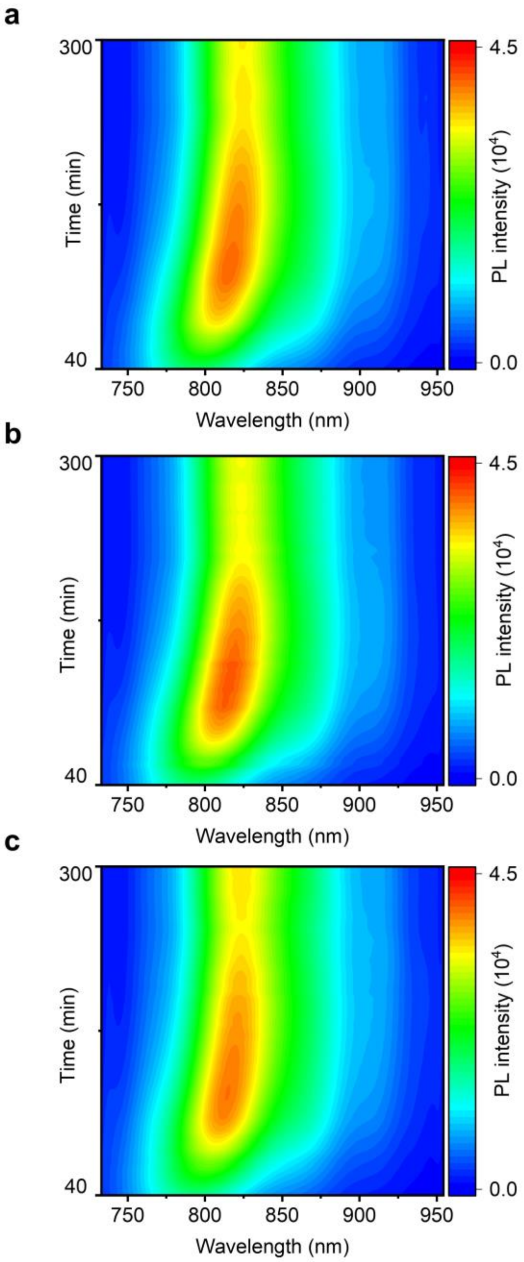

Supplementary Fig. 12. The in-situ PL spectra of the three polymerization reactions (**a**, **b**, and **c**) of PYT under the same reaction conditions over 300 minutes.

Supplementary Table 4. The GPC results of each set of batches in the Stille polymerization vary the reaction times.

Reaction Time (min)	D-monomer batch	A-monomer batch	Catalyst	M_n (kDa)	M_w (kDa)	*Đ*
45	1	δ	Supplier_1A	5.7	6.9	1.2
70	1	δ	Supplier_1A	7.0	9.5	1.3
100	1	δ	Supplier_1A	7.4	9.8	1.3
120	1	δ	Supplier_1A	7.6	10.6	1.4
140	1	δ	Supplier_1A	8.3	12.2	1.5
160	1	δ	Supplier_1A	8.4	13.2	1.6
180	1	δ	Supplier_1A	8.1	14.2	1.8
195	1	δ	Supplier_1A	9.1	15.7	1.7
220	1	δ	Supplier_1A	10.1	17.6	1.7
250	1	δ	Supplier_1A	10.1	18.9	1.9
270	1	δ	Supplier_1A	10.9	20.7	1.9
300	1	δ	Supplier_1A	10.7	21.5	2.0

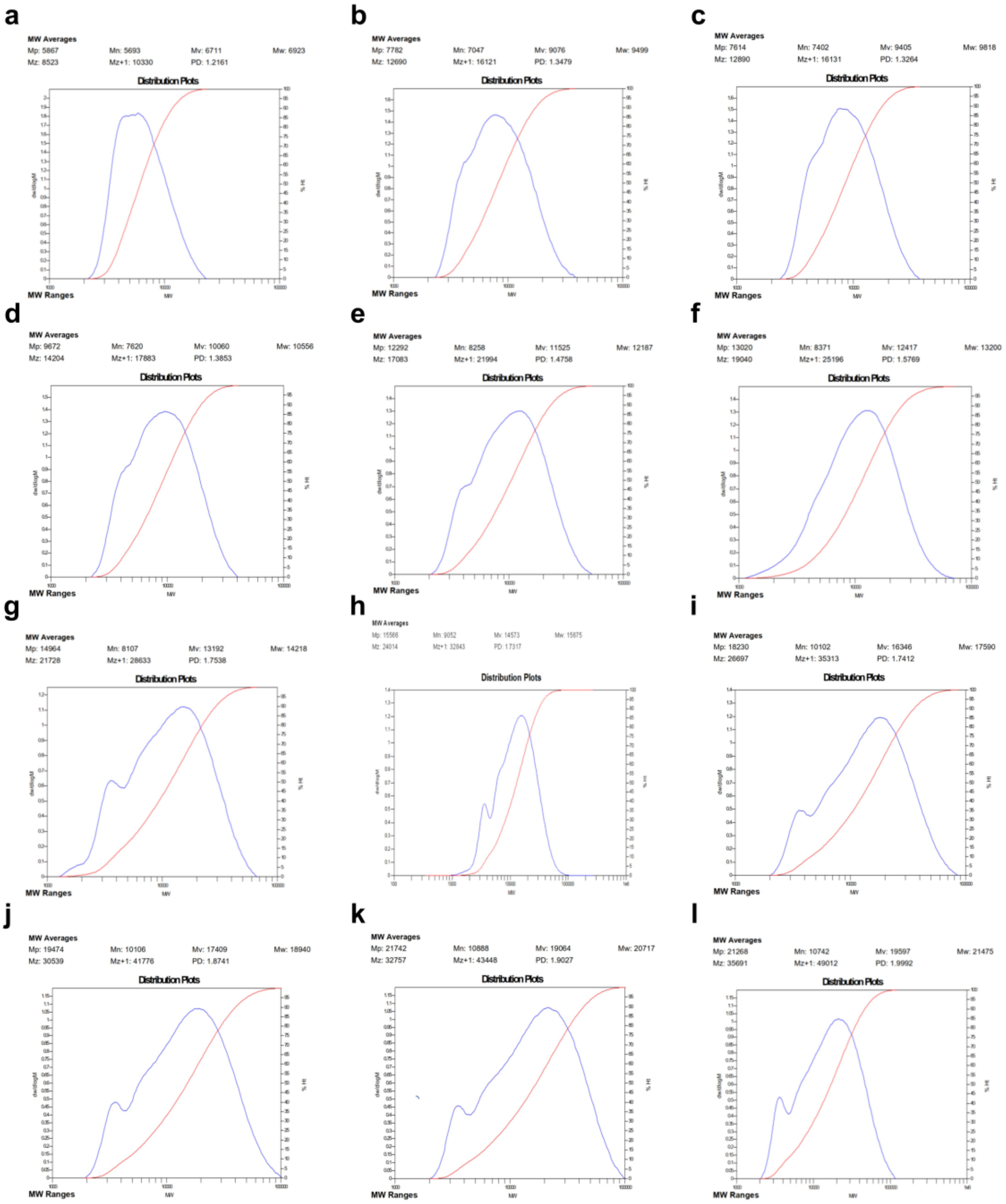

Supplementary Fig. 13. The GPC curves of each set of batches in the Stille polymerization varying the reaction times, including **a** 45 mins, **b** 70 mins, **c** 100 mins, **d** 120 mins, **e** 140 mins, **f** 160 mins, **g** 180 mins, **h** 195 mins, **i** 220 mins, **j** 250 mins, **k** 270 mins, **l** 300 mins.

Supplementary Table 5. The photovoltaic parameters of the PBDB-T:PYT all-polymer systems fabricated by different PYT batches, were measured under one sun illumination.

M_w (kDa)	V_{OC} (V)	J_{SC} (mA cm^{-2})	FF (%)	PCE (PCE^a) (%)
6.9	0.850	22.92	63.56	12.38 (12.13)
9.5	0.858	23.38	64.14	12.87 (12.65)
9.8	0.869	22.29	67.67	13.12 (12.85)
10.6	0.859	23.12	68.66	13.63 (13.41)
12.2	0.863	23.53	69.37	14.09 (13.78)
13.2	0.868	23.52	71.13	14.52 (14.31)
14.2	0.885	23.62	70.20	14.68 (14.43)
15.7	0.877	23.44	69.66	14.31 (14.09)
17.6	0.868	23.14	66.95	13.45 (13.25)
18.9	0.872	23.06	66.89	13.46 (13.24)
20.7	0.857	22.47	66.15	12.74 (12.47)
21.5	0.857	22.62	65.87	12.77 (12.50)

[a] The statistics were obtained from over six devices.

Supplementary Table 6. The photovoltaic parameters of the PM6:PYT all-polymer systems fabricated by different PYT batches, were measured under one sun illumination.

M_w (kDa)	V_{OC} (V)	J_{SC} (mA cm^{-2})	FF (%)	PCE (PCE[a]) (%)
6.9	0.950	21.41	63.01	12.81 (12.41)
9.5	0.934	21.82	64.33	13.12 (12.98)
9.8	0.933	22.86	65.93	14.07 (13.86)
10.6	0.943	21.26	64.22	12.87 (12.56)
12.2	0.953	20.07	64.95	12.42 (12.21)
13.2	0.951	19.01	68.18	12.32 (12.01)
14.2	0.950	18.99	63.98	11.43 (11.20)
15.7	0.956	19.13	62.90	11.46 (11.24)
17.6	0.945	18.31	65.20	11.28 (11.05)
18.9	0.947	17.27	62.42	10.21 (10.04)
20.7	0.944	17.55	63.02	10.44 (10.22)
21.5	0.952	17.71	62.42	10.52 (10.34)

[a] The statistics were obtained from over six devices.

Supplementary Table 7. The photovoltaic parameters of the PM7:PYT all-polymer systems fabricated by different PYT batches, were measured under one sun illumination.

M_w (kDa)	V_{OC} (V)	J_{SC} (mA cm^{-2})	FF (%)	PCE (PCE[a]) (%)
6.9	0.918	14.82	56.06	7.62 (7.47)
9.5	0.959	14.11	55.15	7.47 (7.18)
9.8	0.969	13.90	57.94	7.81 (7.52)
10.6	0.952	17.84	49.32	8.38 (8.04)
12.2	0.983	16.60	55.38	9.03 (8.81)
13.2	0.980	17.25	57.07	9.65 (9.27)
14.2	0.965	16.31	54.37	8.57 (8.23)
15.7	0.969	14.19	56.63	7.79 (7.49)
17.6	0.970	13.49	53.94	7.06 (6.78)
18.9	0.949	16.67	48.01	7.60 (7.41)
20.7	0.958	13.81	49.62	6.56 (6.23)
21.5	0.974	11.25	55.31	6.06 (5.84)

[a] The statistics were obtained from over six devices.

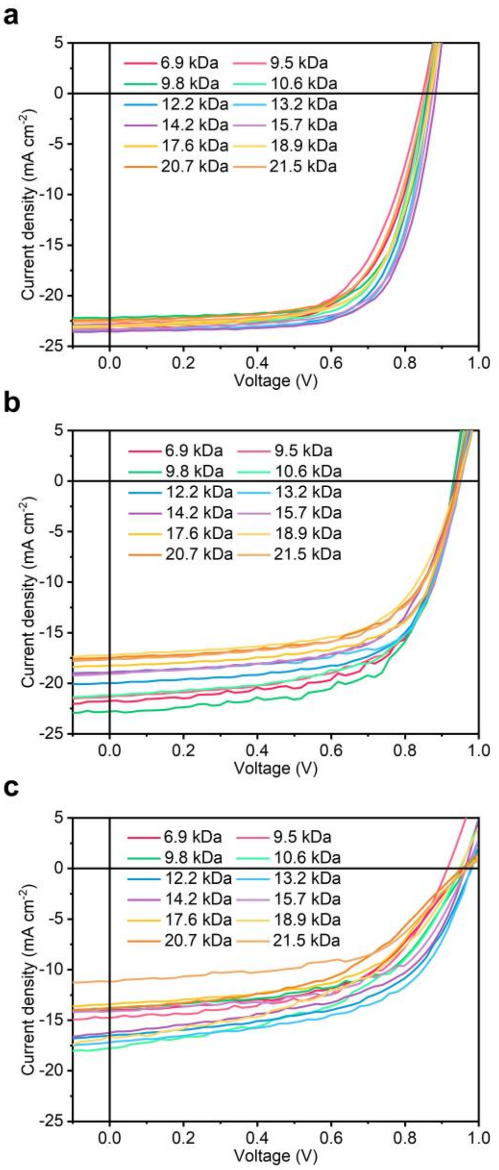

Supplementary Fig. 14. The relevant *J-V* curves of the **a** PBDB-T:PYT, **b** PM6:PYT and **c** PM7:PYT all-polymer systems fabricated by different PYT batches, measured under one sun illumination.

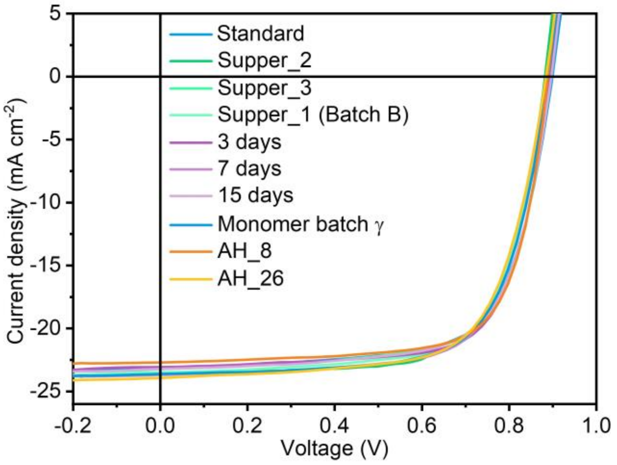

Supplementary Fig. 15. The relevant *J-V* curves of the PBDB-T:PYT all-polymer systems fabricated by different PYT batches, were measured under one sun illumination.

Supplementary Table 8. GPC parameters for PYT polymer batches.

Batches	D-monomer batch	A-monomer batch	Catalyst	Exposed Time (day)	AH (g m^{-3})	M_n (kDa)	M_w (kDa)	*Đ*
Standard	1	δ	Supplier_1 (Batch A)	0	~14	8.1	14.2	1.8
A	1	δ	Supplier_2	0	~14	7.5	13.8	1.8
B	1	δ	Supplier_3	0	~14	7.5	14.0	1.9
C	1	δ	Supplier_1 (Batch B)	0	~14	8.6	14.9	1.7
D	1	δ	Supplier_1 (Batch A)	3	~14	7.3	13.8	1.9
E	1	δ	Supplier_1 (Batch A)	7	~14	7.4	14.2	1.9
F	1	δ	Supplier_1 (Batch A)	15	~14	7.3	14.9	2.0
G	1	γ	Supplier_1 (Batch A)	0	~14	9.2	14.9	1.6
H	1	δ	Supplier_1 (Batch A)	0	~8	8.4	15.0	1.8
I	1	δ	Supplier_1 (Batch A)	0	~26	8.4	14.8	1.8

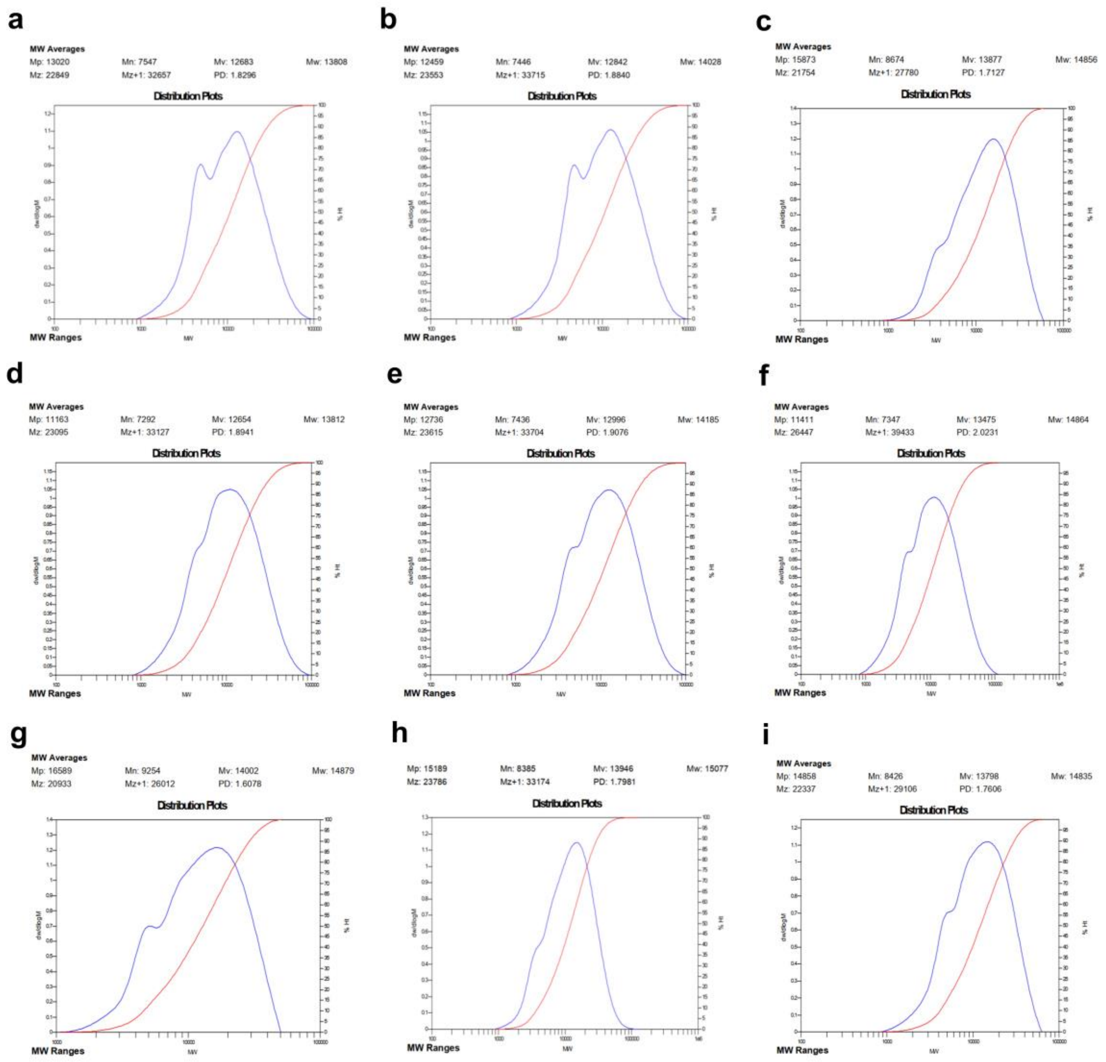

Supplementary Fig. 16. GPC profiles for the synthesized PYT polymers from batches **a** to **i**.

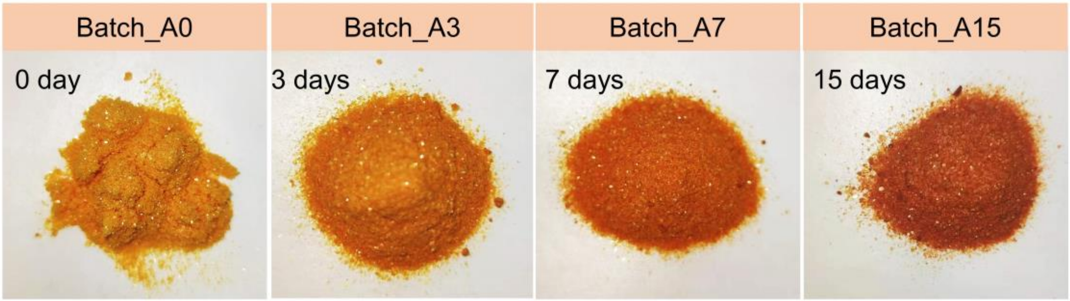

Supplementary Fig. 17. Pictures of the $Pd(PPh_3)_4$ catalyst Batch_1 (or namely Batch_A0) with various aging periods.

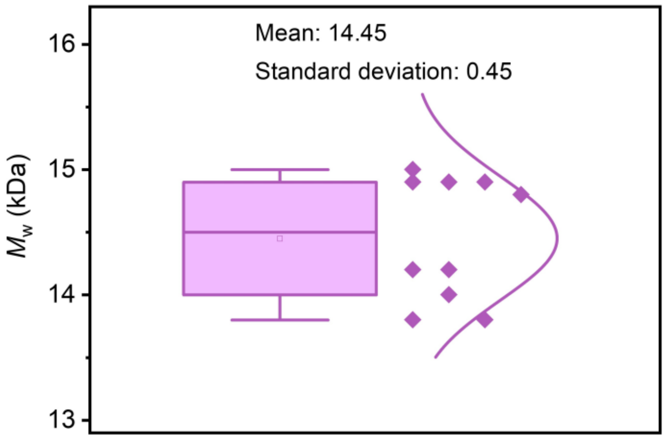

Supplementary Fig. 18. M_w statistical analysis diagram of PYT.

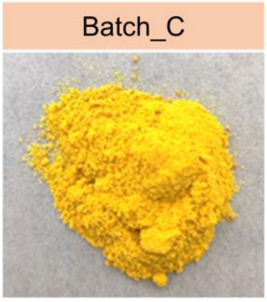

Supplementary Fig. 19. Pictures of the $Pd(PPh_3)_4$ catalyst Batch_C (Supplier_1) which was used in universality studies.

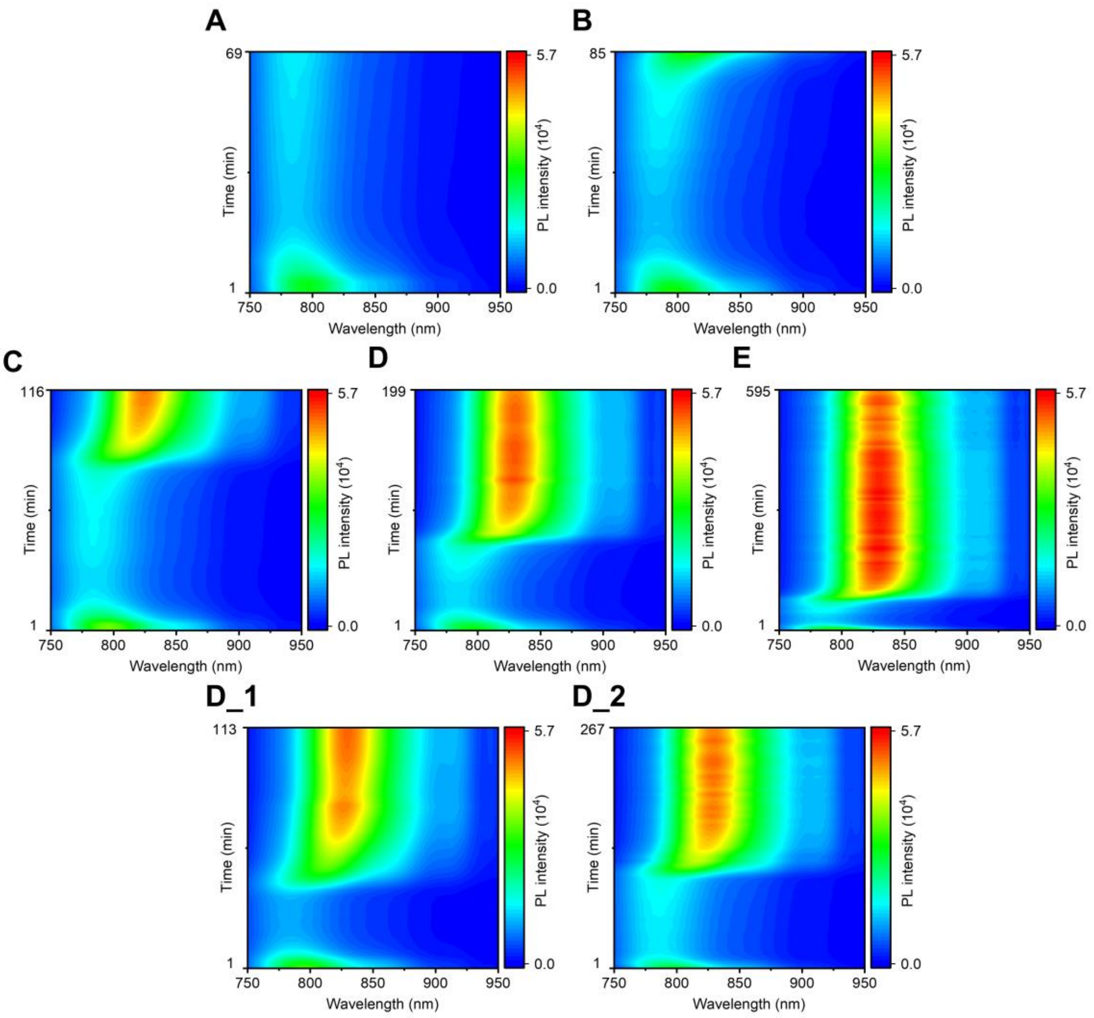

Supplementary Fig. 20. Two-dimensional PL spectrum maps of the PY-IT polymerization process for batches A to D_2.

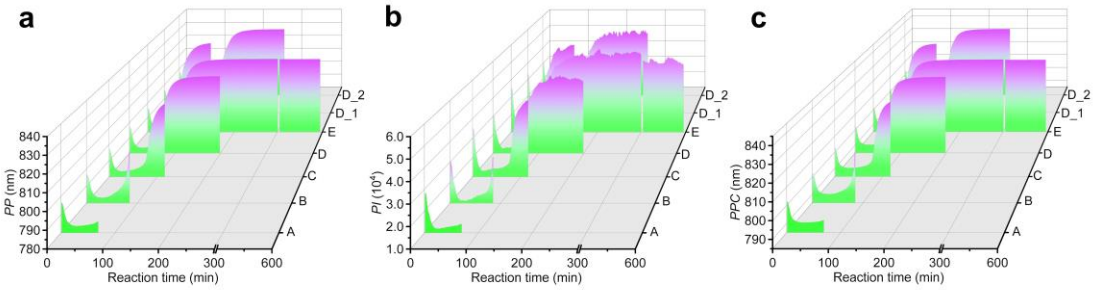

Supplementary Fig. 21. The PY-IT three-dimensional trends of **a** *PP*, **b** *PI*, and **c** *PPC* vary with the polymerization time.

Supplementary Table 9. GPC parameters for PY-IT polymer batches.

Batch	Catalyst amount	Reaction time (min)	M_n (kDa)	M_w (kDa)	$Đ$
A	1.00 times	69	4.9	5.7	1.2
B	1.00 times	85	5.9	8.9	1.5
C	1.00 times	116	6.2	9.4	1.3
D	1.00 times	199	7.6	11.9	1.5
E	1.00 times	595	8.2	19.0	2.3
D_1	1.33 times	113	8.5	12.4	1.5
D_2	0.67 times	267	7.2	11.5	1.6

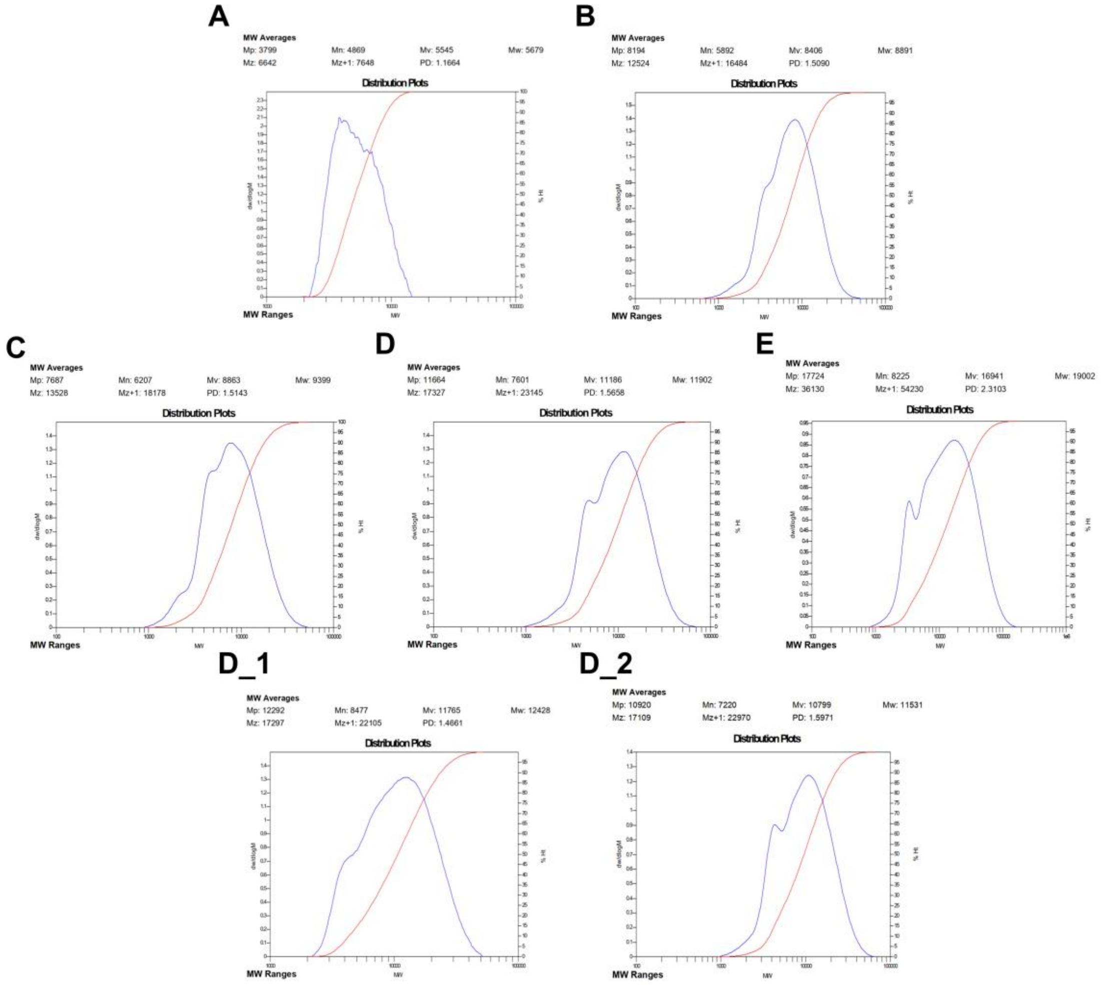

Supplementary Fig. 22. GPC profiles for the synthesized PY-IT polymers from batches A to D_2.

Supplementary Table 10. The photovoltaic parameters of the PM6:PY-IT all-polymer systems fabricated by different PY-IT batches, were measured under one sun illumination.

Batch	M_w (kDa)	V_{OC} (V)	J_{SC} (mA cm^{-2})	FF (%)	PCE (PCE[a]) (%)
A	5.7	0.852	23.00	58.58	11.49 (11.32)
B	8.9	0.927	22.87	57.22	12.13 (11.95)
C	9.4	0.929	22.81	62.36	13.21 (13.00)
D	11.9	0.943	22.92	71.85	15.53 (15.16)
E	19.0	0.937	22.95	67.85	14.59 (14.34)
D_1	12.4	0.941	22.89	71.70	15.44 (15.29)
D_2	11.5	0.940	22.89	71.65	15.42 (15.26)

[a] The statistics were obtained from over six devices.

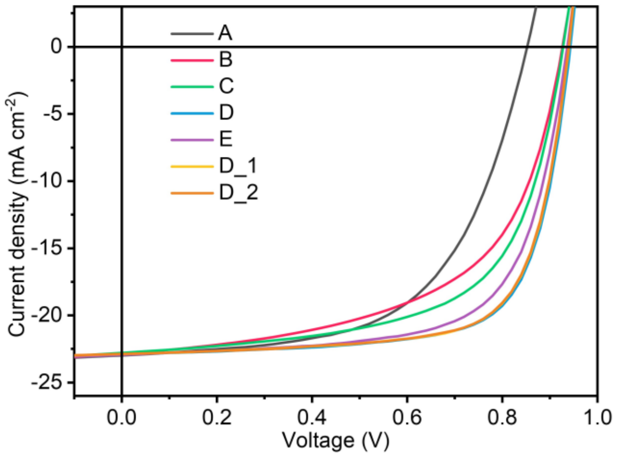

Supplementary Fig. 23. The relevant *J*-*V* curves of the PM6:PY-IT all-polymer systems fabricated by different PY-IT batches, were measured under one sun illumination.

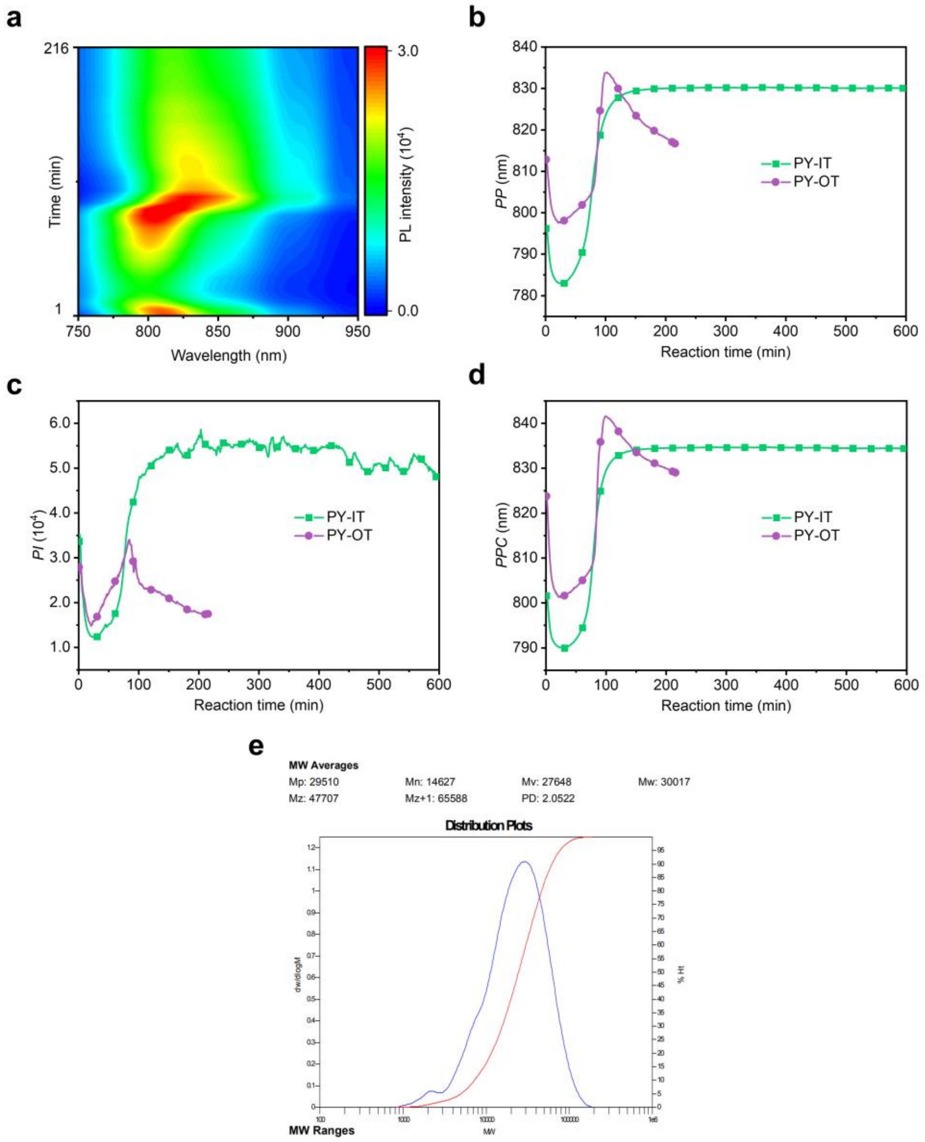

Supplementary Fig. 24. **a** Two-dimensional PL spectrum maps of the PY-IT synthesis. The traces of **b** *PP*, **c** *PI* and **d** *PPC* of PY-OT and PY-IT polymerization reactions. **e** GPC profiles of the synthesized PY-OT.

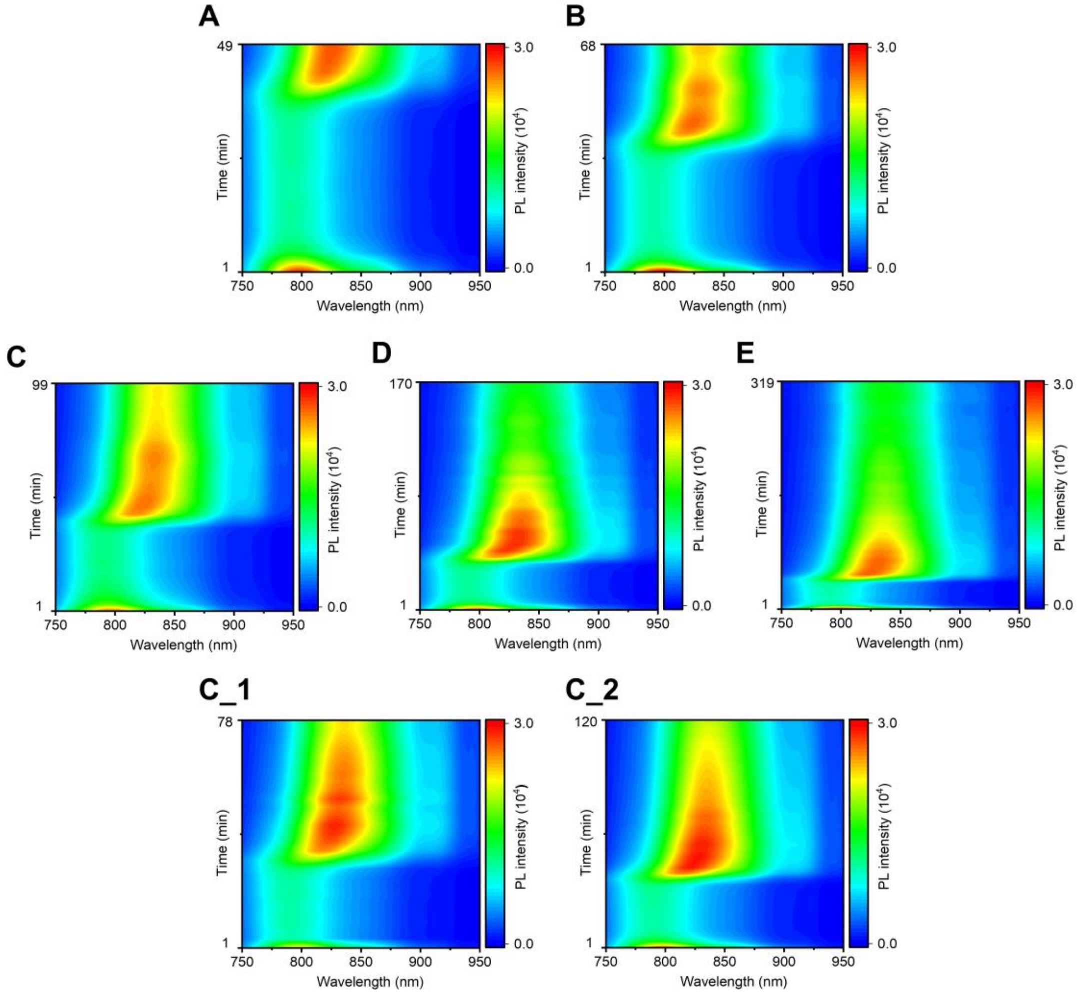

Supplementary Fig. 25. Two-dimensional PL spectrum maps of the PYF-T-*o* polymerization process for batches A to C_2.

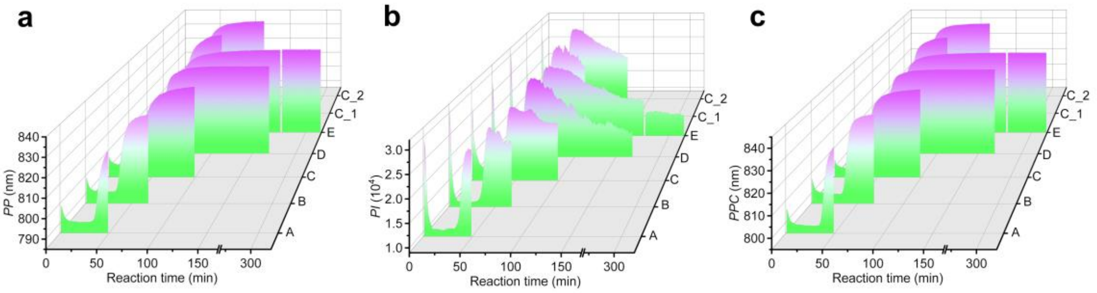

Supplementary Fig. 26. The three-dimensional trends of **a** *PP*, **b** *PI*, and **c** *PPC* vary with the polymerization time for PYF-T-*o*.

Supplementary Table 11. GPC parameters for PYF-T-*o* polymer batches.

Batch	Catalyst amount	Reaction time (min)	M_n (kDa)	M_w (kDa)	$Đ$
A	1.00 times	49	4.9	7.3	1.5
B	1.00 times	68	6.3	9.6	1.5
C	1.00 times	99	6.4	10.1	1.6
D	1.00 times	170	9.5	18.7	2.0
E	1.00 times	319	9.8	20.7	2.1
C_1	1.33 times	78	6.7	10.9	1.6
C_2	0.67 times	120	6.7	10.5	1.6

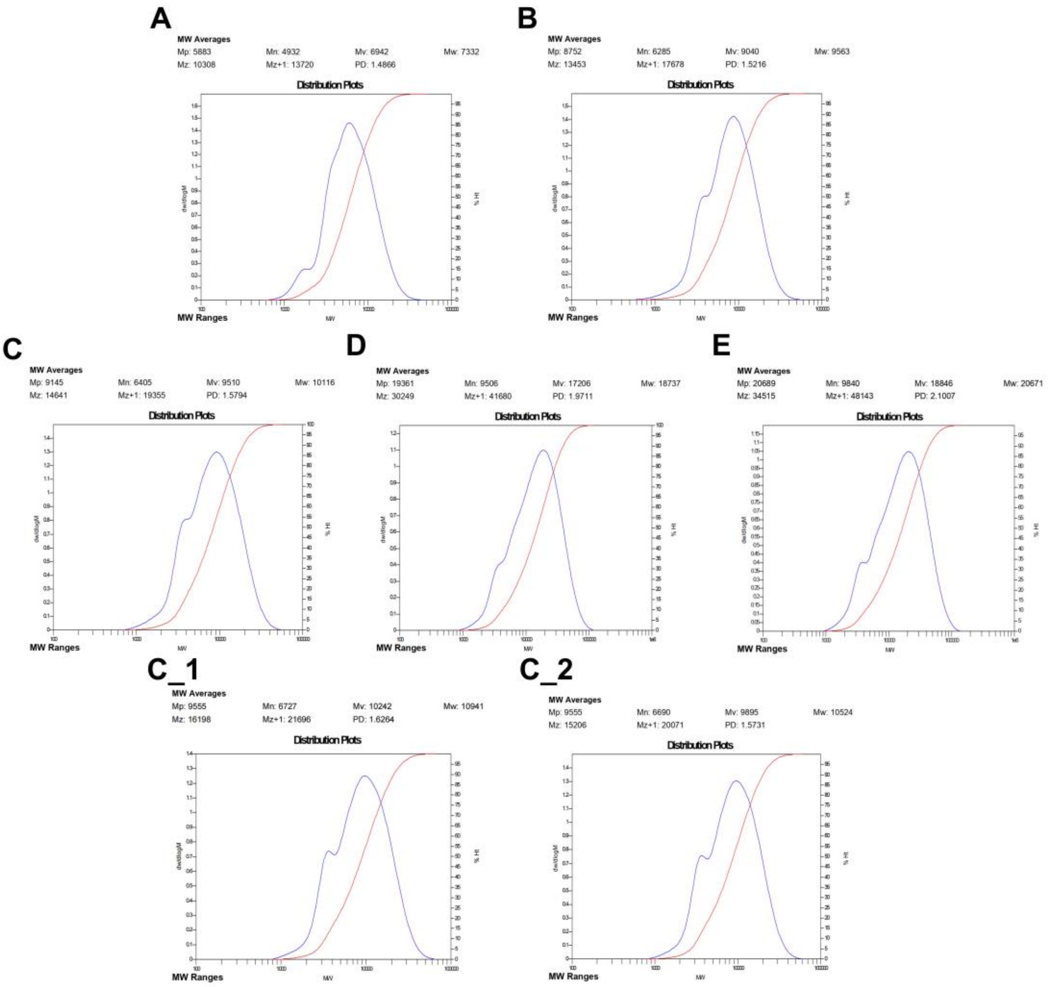

Supplementary Fig. 27. GPC profiles for the synthesized PYF-T-*o* polymers from batches A to C_2.

Supplementary Table 12. The photovoltaic parameters of the PM6:PYF-T-*o* all-polymer systems fabricated by different PYF-T-*o* batches, were measured under one sun illumination.

Batch	M_w (kDa)	V_{OC} (V)	J_{SC} (mA cm^{-2})	FF (%)	PCE (PCE[a]) (%)
A	7.3	0.875	20.09	45.02	7.91 (7.77)
B	9.6	0.883	23.00	51.79	10.52 (10.27)
C	10.1	0.892	23.52	58.11	12.20 (11.99)
D	18.7	0.895	23.66	57.02	12.08 (11.85)
E	20.7	0.887	23.32	57.43	11.89 (11.74)
C_1	10.9	0.892	23.88	56.09	11.95 (11.81)
C_2	10.5	0.886	23.95	56.00	11.88 (11.78)

[a] The statistics were obtained from over six devices.

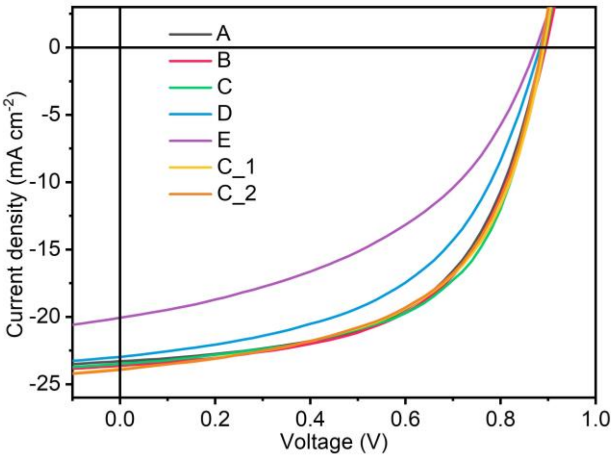

Supplementary Fig. 28. The relevant *J*-*V* curves of the PM6:PYF-T-*o* all-polymer systems fabricated by different PYF-T-*o* batches, were measured under one sun illumination.

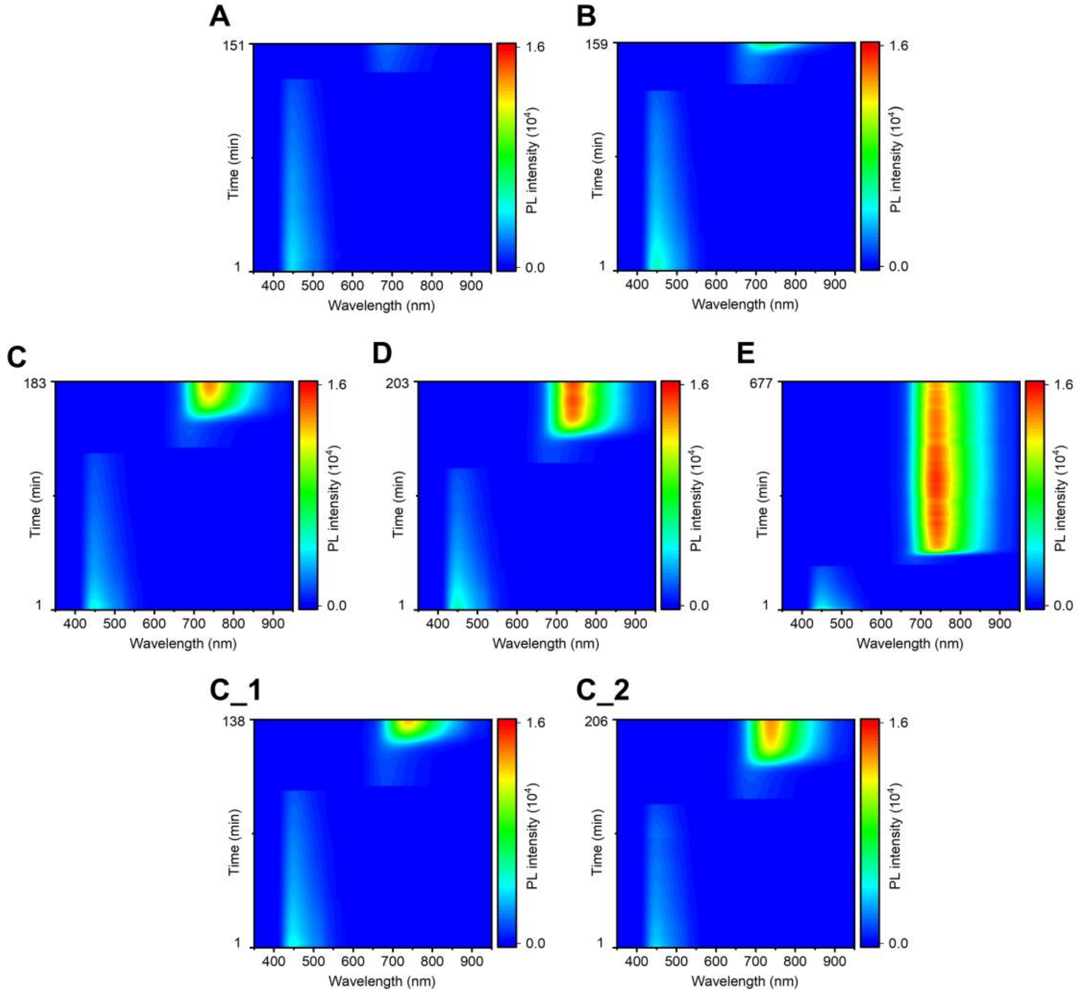

Supplementary Fig. 29. Two-dimensional PL spectrum maps of the PTIB polymerization process for batches A to C_2.

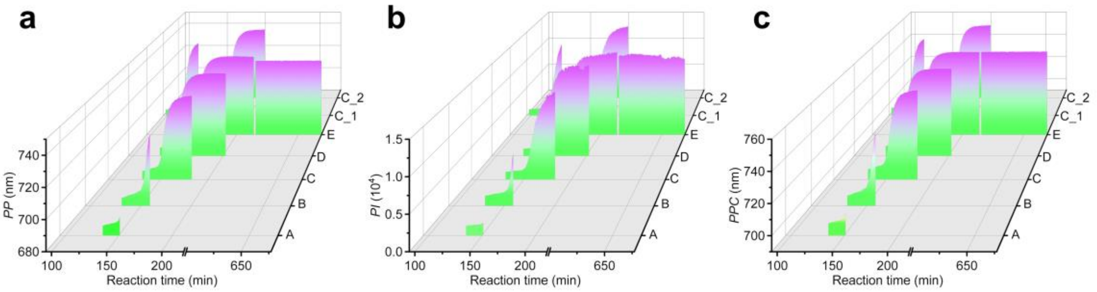

Supplementary Fig. 30. The three-dimensional trends of **a** *PP*, **b** *PI*, and **c** *PPC* vary with the polymerization time for PTIB.

Supplementary Table 13. GPC parameters for PTIB polymer batches.

Batch	Catalyst amount	Reaction time (min)	M_n (kDa)	M_w (kDa)	*Đ*
A	1.00 times	151	3.0	3.6	1.2
B	1.00 times	159	4.1	5.8	1.4
C	1.00 times	183	6.5	9.3	1.4
D	1.00 times	203	8.0	13.2	1.7
E	1.00 times	677	11.0	23.3	2.1
C_1	1.33 times	138	6.8	9.4	1.4
C_2	0.67 times	206	5.8	8.8	1.5

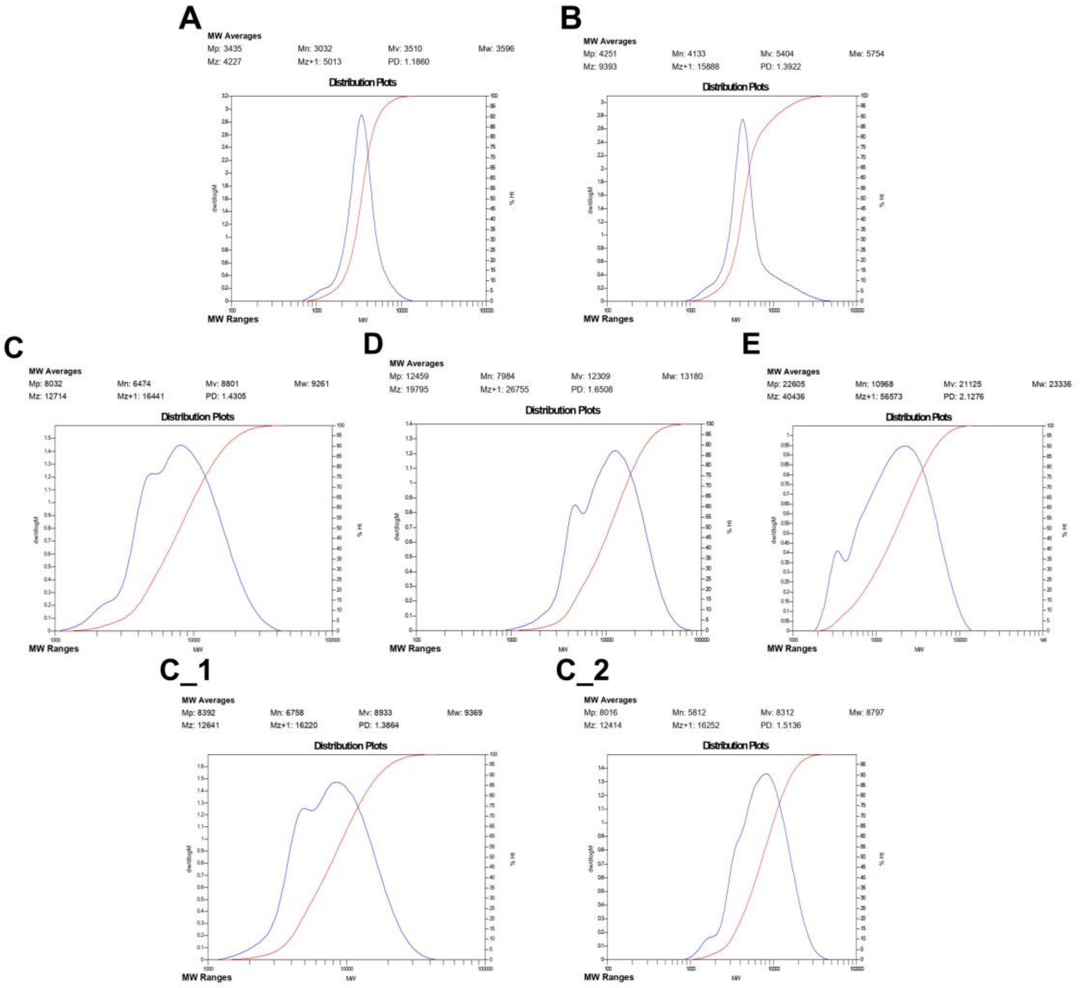

Supplementary Fig. 31. GPC profiles for the synthesized PTIB polymers from batches A to C_2.

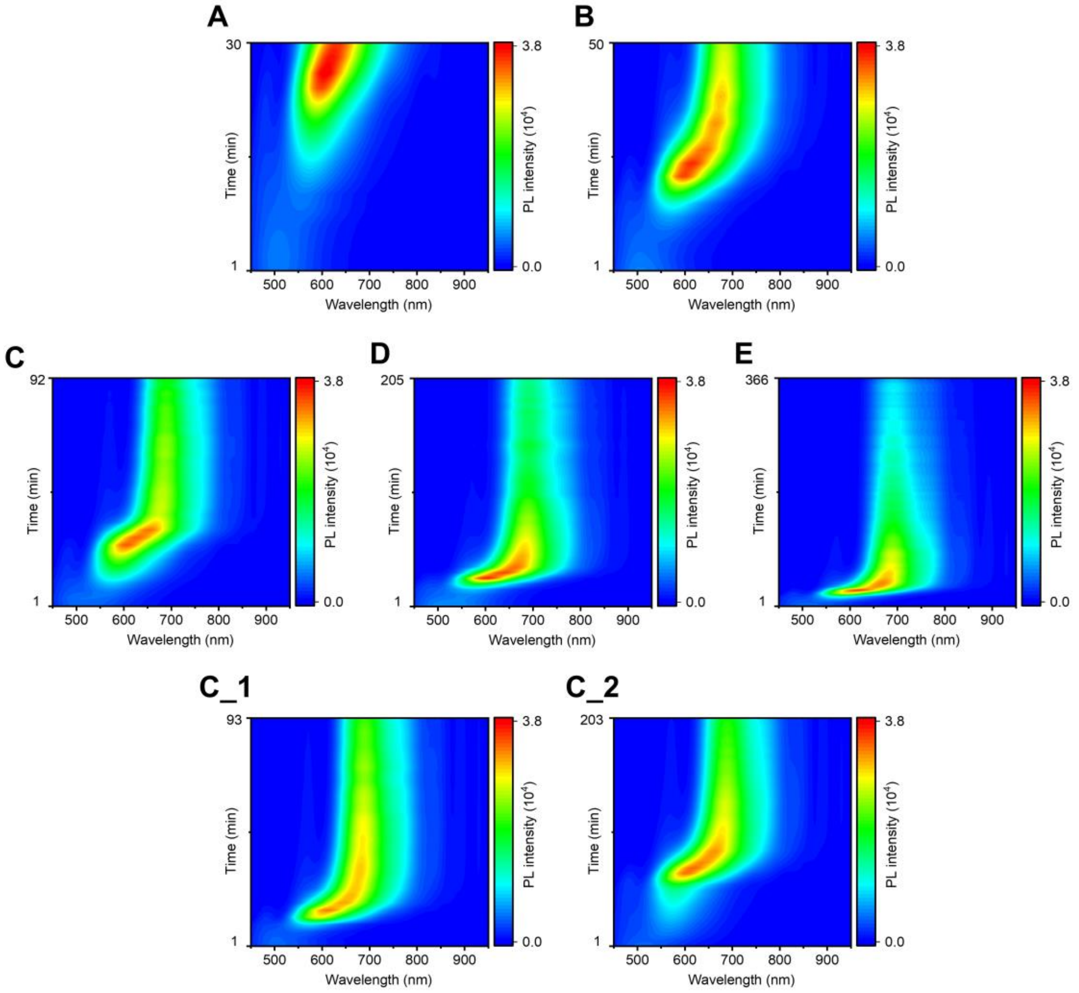

Supplementary Fig. 32. Two-dimensional PL spectrum maps of the PM6 polymerization process for batches A to C_2.

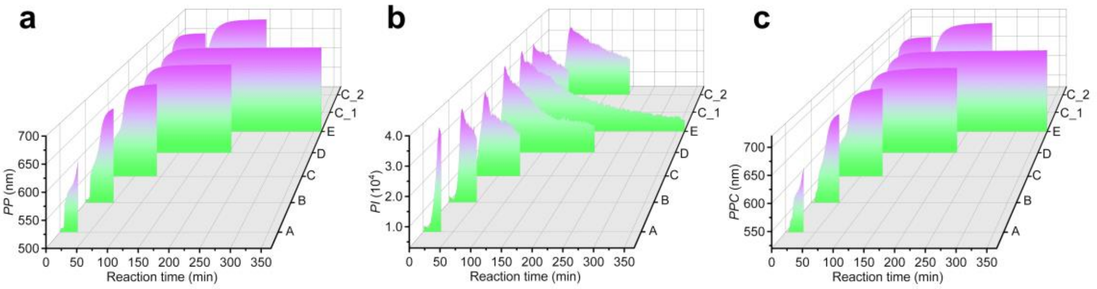

Supplementary Fig. 33. The three-dimensional trends of **a** *PP*, **b** *PI*, and **c** *PPC* vary with the polymerization time for PM6.

Supplementary Table 14. GPC parameters for PM6 polymer batches.

Batch	Catalyst amount	Reaction time (min)	M_n (kDa)	M_w (kDa)	$Đ$
A	1.00 times	30	4.4	8.7	2.0
B	1.00 times	50	8.0	19.4	2.4
C	1.00 times	92	18.1	39.7	2.2
D	1.00 times	205	19.2	50.0	2.6
E	1.00 times	366	29.3	57.6	2.0
C_1	1.33 times	93	18.9	39.0	2.1
C_2	0.67 times	203	19.9	42.2	2.1

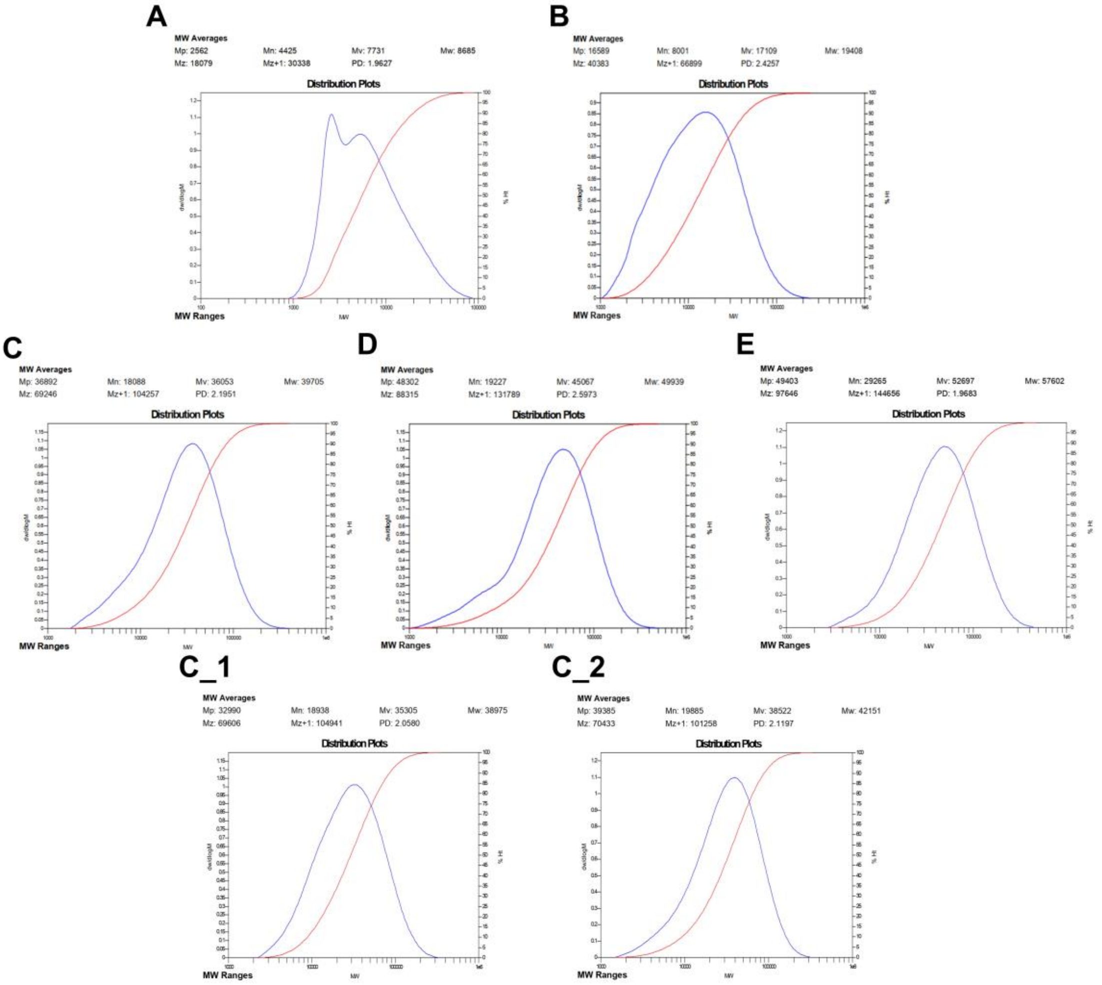

Supplementary Fig. 34. GPC profiles for the synthesized PM6 polymers from batches A to C_2.

Supplementary Table 15. The photovoltaic parameters of the PM6:Y6 systems fabricated different PM6 batches as the additive, which were blade-coated at a rate of 30 m min^{-1} in the air from a solution of PM6:Y6 (1:1.2, with 5 wt% PM6) with 6.0 mg mL^{-1} solution concentrations in chloroform.

Batch	M_w (kDa)	V_{OC} (V)	J_{SC} (mA cm^{-2})	FF (%)	PCE (PCE^a) (%)
A	8.7	0.865	24.27	71.86	15.11 (15.02)
B	19.4	0.865	24.51	72.32	15.35 (15.20)
C	39.7	0.850	25.54	74.36	16.10 (15.98)
D	50.0	0.852	25.39	72.73	15.74 (15.58)
E	57.6	0.862	23.96	68.61	14.19 (14.13)
C_1	39.0	0.856	25.04	73.78	15.81 (15.67)
C_2	42.2	0.852	24.65	73.48	15.57 (15.41)

[a] The statistics were obtained from over six devices.

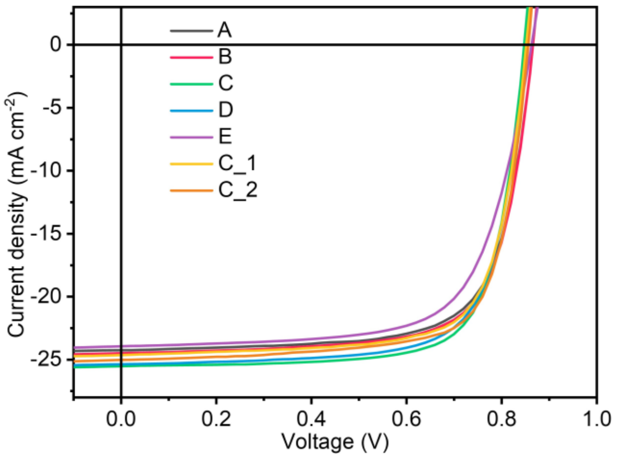

Supplementary Fig. 35. The relevant *J*-*V* curves of the PM6:Y6 systems fabricated by different PM6 batches as the additive, which were blade-coated at a rate of 30 m min^{-1} in the air from a solution of PM6:Y6 (1:1.2, with 5 wt% PM6 which was synthesized in this work) with 6.0 mg mL^{-1} solution concentrations in chloroform.

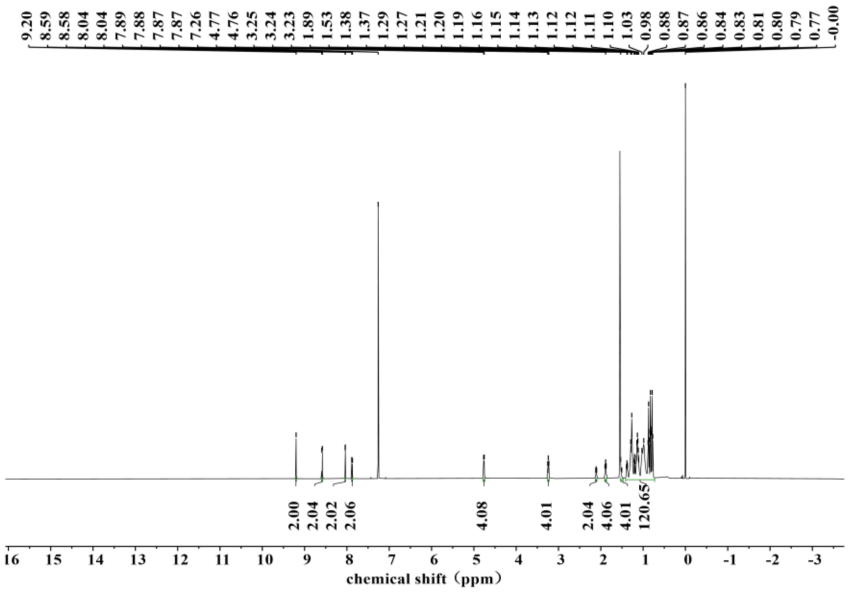

Supplementary Fig. 36. ^{1}H NMR spectrum of PY-IT monomer Y5-C20-Br-γ.

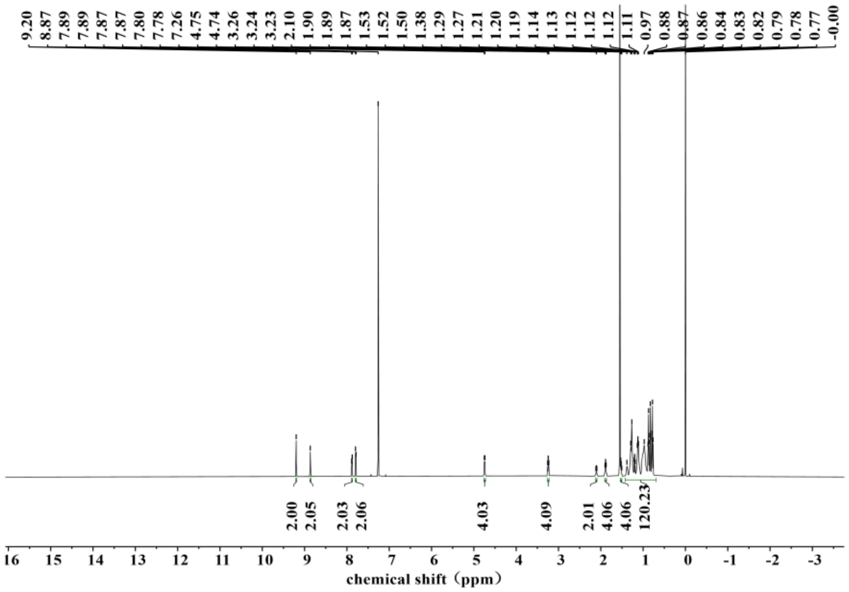

Supplementary Fig. 37. ^{1}H NMR spectrum of PY-OT monomer Y5-C20-Br-δ.

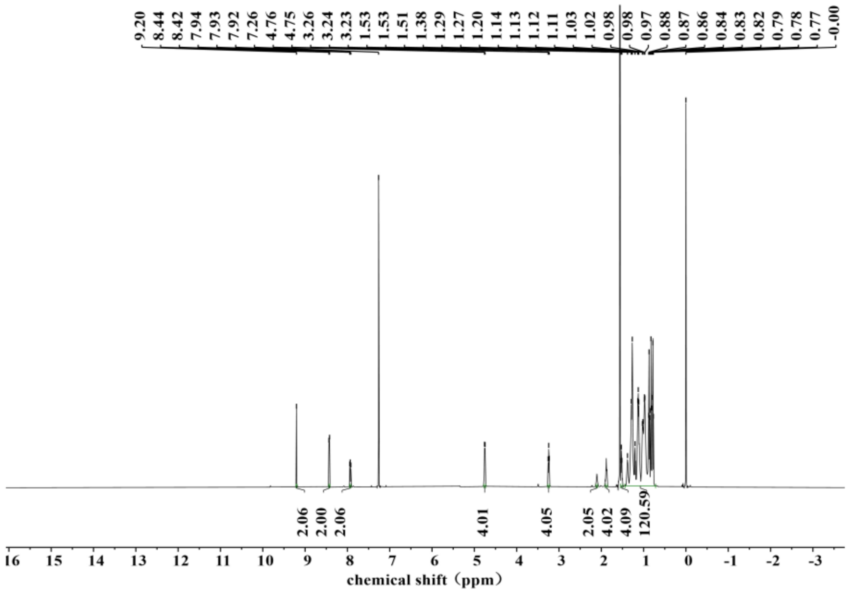

Supplementary Fig. 38. ^{1}H NMR spectrum of PYF-T-*o* monomer Y5-OD-FBr-*o*.

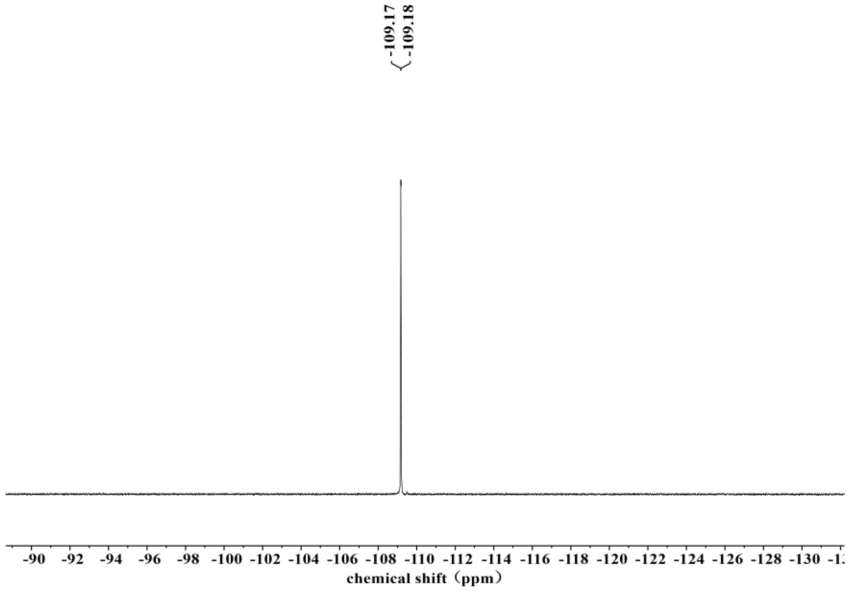

Supplementary Fig.39. ^{19}F NMR spectrum of PYF-T-*o* monomer Y5-OD-FBr-*o*.

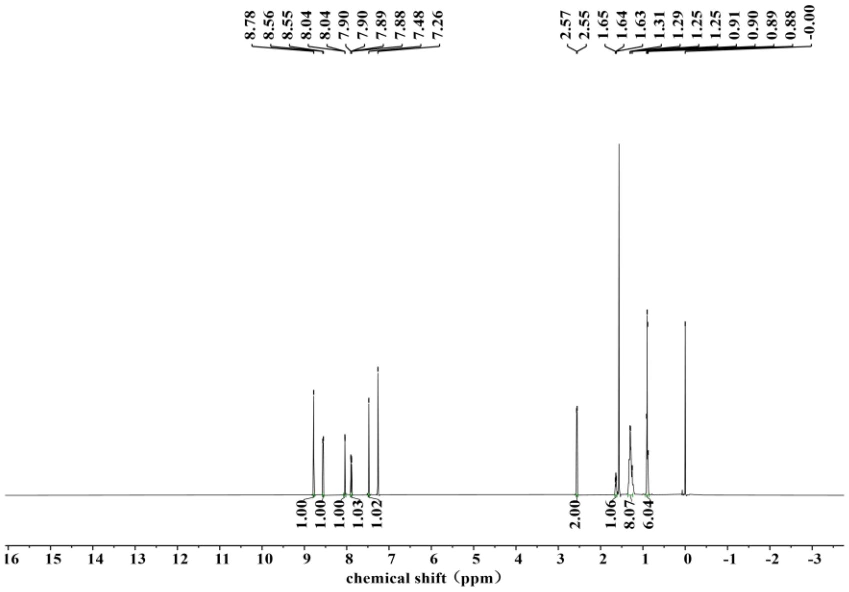

Supplementary Fig. 40. ^{1}H NMR spectrum of PTIB monomer TIC-Br.

References

1. Cai Y., *et al.* Improved molecular ordering in a ternary blend enables all-polymer solar cells over 18% efficiency. *Adv. Mater.* **35**, 2208165 (2023).

2. Sun R., *et al.* 18.2%-efficient ternary all-polymer organic solar cells with improved stability enabled by a chlorinated guest polymer acceptor. *Joule* **7**, 221-237 (2023).

3. Yang X., *et al.* Ternary all-polymer solar cells with efficiency up to 18.14% employing a two-step sequential deposition. *Adv. Mater.* **35**, 2209350 (2023).

4. Qiu J., *et al.* Linear regulating of polymer acceptor aggregation with short alkyl chain units enhances all-polymer solar cells' efficiency. *Macromol. Rapid Commun.* **44**, 2200753 (2023).

5. Li Y., *et al.* Polymerized small molecular acceptor with branched side chains for all polymer solar cells with eficiency over 16.7%. *Adv. Mater.* **34**, 2110155 (2022).

6. Zhang J., *et al.* π‐Extended conjugated polymer acceptor containing thienylene‐vinylene‐thienylene unit for high‐performance thick‐film all‐polymer solar cells with superior long‐term stability. *Adv. Energy Mater.* **11**, 2102559 (2021).

7. Zhao F., Zhou J., He D., Wang C., Lin Y. Low-cost materials for organic solar cells. *J. Mater. Chem. C* **9**, 15395-15406 (2021).

8. Fu H., *et al.* High efficiency (15.8%) all-polymer solar cells enabled by a regioregular narrow bandgap polymer acceptor. *J. Am. Chem. Soc.* **143**, 2665-2670 (2021).

9. Xian K., *et al.* Simultaneous optimization of efficiency, stretchability, and stability in all‐polymer solar cells via aggregation control. *Chin. J. Chem.* **41**, 159-166 (2022).

10. Cao C., *et al.* Quasiplanar heterojunction all‐polymer solar cells: A dual approach to stability. *Adv. Funct. Mater.* **32**, 2201828 (2022).

11. Wang T., Sun R., Wu Y., Wang W., Zhang M., Min J. Constructing a double-cable polymer acceptor for efficient all-polymer solar cells with a non-

radiative recombination energy loss of 0.16 eV. *Chem. Mater.* **34**, 9970-9981 (2022).

12. Zhou D., *et al.* Binary blend all-polymer solar cells with a record efficiency of 17.41% enabled by programmed fluorination both on donor and acceptor blocks. *Adv. Sci.* **9**, 2202022 (2022).

13. Fan Q., *et al.* Near-infrared absorbing polymer acceptors enabled by selenophene-fused core and halogenated end-group for binary all-polymer solar cells with efficiency over 16%. *Nano Energy* **92**, 106718 (2022).

14. Chen D., *et al.* Rational regulation of the molecular aggregation enables a facile blade-coating process of large-area all-polymer solar cells with record efficiency. *Small* **18**, 2200734 (2022).

15. Fu H., Li Y., Wu Z., Lin F. R., Woo H. Y., Jen A. K. Side-chain substituents on benzotriazole-based polymer acceptors affecting the performance of all-polymer solar cells. *Macromol. Rapid Commun.* **43**, 2200062 (2022).

16. Sun C., *et al.* Synergistic engineering of side chains and backbone regioregularity of polymer acceptors for high‐performance all‐polymer solar cells with 15.1% efficiency. *Adv. Energy Mater.* **12**, 2103239 (2021).

17. Ren J., *et al.* Molecular design revitalizes the low-cost PTV-polymer for highly efficient organic solar cells. *Natl. Sci. Rev.* **8**, nwab031 (2021).

18. Su N., *et al.* High-efficiency all-polymer solar cells with poly-small-molecule acceptors having π-extended units with broad near-IR absorption. *ACS Energy Lett.* **6**, 728-738 (2021).

19. Fan Q., *et al.* Over 14% efficiency all-polymer solar cells enabled by a low bandgap polymer acceptor with low energy loss and efficient charge separation. *Energy Environ. Sci.* **13**, 5017-5027 (2020).

20. Wang W., *et al.* Controlling molecular mass of low-band-gap polymer acceptors for high-performance all-polymer solar cells. *Joule* **4**, 1070-1086 (2020).

21. Wu Y., *et al.* Fine-tuning semiconducting polymer self-aggregation and crystallinity enables optimal morphology and high-performance printed all-polymer solar cells. *J Am. Chem. Soc.* **142**, 392-406 (2020).

22. Zhu L., *et al.* Aggregation-induced multilength scaled morphology enabling 11.76% efficiency in all-polymer solar cells using printing fabrication. *Adv. Mater.* **31**, 1902899 (2019).

23. Zhang Z. G., *et al.* Constructing a strongly absorbing low-bandgap polymer acceptor for high-performance all-polymer solar cells. *Angew. Chem. Int. Ed.* **56**, 13503-13507 (2017).

24. Zhao R., Wang N., Yu Y., Liu J. Organoboron polymer for 10% Efficiency all-polymer solar cells. *Chem. Mater.* **32**, 1308-1314 (2020).

25. Li Y., *et al.* 8.78% Efficient all-polymer solar cells enabled by polymer acceptors based on a B<--N embedded electron-deficient unit. *Adv. Mater.* **31**, 1904585 (2019).
