# Supplementary material for: Real-time monitoring polymerization degree of organic photovoltaic materials toward no batch-to-batch variations in device performance
Source: Nat Commun. 2024 Feb 10;15:1248. doi: 10.1038/s41467-024-45510-w (PMC10858892; doi:10.1038/s41467-024-45510-w)
Supplement: Supplementary file 3 — Description of Additional Supplementary Files [file 41467_2024_45510_MOESM3_ESM.pdf]

Description of Additional Supplementary Files

File name: Supplementary Code 1

Description: The code file used in the in-situ PL system contains the source code, a small demonstration dataset and instructions.

File name: Supplementary Movie 1

Description: A Python script runs video, showing the evolution trend of three extracted parameters (including peak position, peak intensity, and peak position at the center of full width at half maximum) of relevant photoluminescence spectra of PYT solution during its polycondensation reactions.
