# Supplementary material for: Real-time monitoring polymerization degree of organic photovoltaic materials toward no batch-to-batch variations in device performance
Source: Nat Commun. 2024 Feb 10;15:1248. doi: 10.1038/s41467-024-45510-w (PMC10858892; doi:10.1038/s41467-024-45510-w)
Supplement: Supplementary file 6 — Reporting Summary [file 41467_2024_45510_MOESM6_ESM.pdf]

nature portfolio

Corresponding author(s): Jie Min

Solar Cells Reporting Summary

Nature Portfolio wishes to improve the reproducibility of the work that we publish. This form is intended for publication with all accepted papers reporting the characterization of photovoltaic devices and provides structure for consistency and transparency in reporting. Some list items might not apply to an individual manuscript, but all fields must be completed for clarity.

For further information on Nature Research policies, including our data availability policy, see Authors & Referees.

Experimental design

Please check the following details are reported in the manuscript, and provide a brief description or explanation where applicable.

1. Dimensions

Item	Yes	No	Description / Explanation
Area of the tested solar cells	☒	☐	Active area of the tested solar cells is 5 mm2 / *Explain why this information is not reported/not relevant.*
Method used to determine the device area	☒	☐	For the solar cells fabricated, the electronic active area of the cell, which was 5 mm2, is defined by the overlap of the ITO electrode and metal electrode. / *Explain why this information is not reported/not relevant.*

2. Current-voltage characterization

Item	Yes	No	Description / Explanation
Current density-voltage (J-V) plots in both forward and backward direction	☐	☒	In general, organic solar cells show the same current density-voltage curves in both forward and backward direction. Thus, we only scan the solar cells in forward direction.
Voltage scan conditions	☒	☐	We scan the solar cells in the range of -0.2-1.2V with a rate of 20 mV s-1. / *Explain why this information is not reported/not relevant.*
Test environment	☒	☐	The measurements were carried out in nitrogen glovebox at room temperature. / *Explain why this information is not reported/not relevant.*
Protocol for preconditioning of the device before its characterization	☒	☐	For the devices measured under one sun illumination, no preconditioning was applied. / *Explain why this information is not reported/not relevant.*
Stability of the J-V characteristic	☐	☒	We did not focus on device stability. / *Explain why this information is not reported/not relevant.*

3. Hysteresis or any other unusual behaviour

Item	Yes	No	Description / Explanation
Description of the unusual behaviour observed during the characterization	☐	☒	*Provide a description of hysteresis or any other unusual behaviour observed during the characterization.* / There is no unusual behaviour observed during the characterization, i.e., hysteresis.
Related experimental data	☐	☒	*Provide a description of the related experimental data.* / We did not find the unusual behaviour.

4. Efficiency

Item	Yes	No	Description / Explanation
External quantum efficiency (EQE) or incident photons to current efficiency (IPCE)	☐	☒	*Provide a description of the technique used.* / We focus on the reproducibility of molecular weight. Therefore we did not test EQE.
A comparison between the integrated response under the standard reference spectrum and the response measure under the simulator	☐	☒	*State where this information can be found in the text.* / We focus on the reproducibility of molecular weight. Therefore we did not test EQE.

For tandem solar cells, the bias illumination and bias voltage used for each subcell

- [ ] Yes — *Provide a description of the measurement conditions.*
- [x] No — We did not make the tandem solar cells in this article.

5. Calibration

Light source and reference cell or sensor used for the characterization

- [x] Yes — An Enli Solar simulator was used as light source and the light intensity was calibrated with a standard single-crystal Si solar cell made by Enli Technology CO., Ltd., Taiwan, calibrated by The National Institute of Metrology (NIM) of China. We show details in the Experimental Section. The J-V curves are measured under simulated AM 1.5 sunlight at 100 mW cm-2 irradiance generated by a xenon lamp.
- [ ] No — *Explain why this information is not reported/not relevant.*

Confirmation that the reference cell was calibrated and certified

- [x] Yes — A standard single-crystal Si solar cell as the reference cell was made by Enli Technology CO., Ltd., and calibrated by The National Institute of Metrology (NIM) of China. This reference cell was used to calibrate the variable intensity under concentrated sun light, with an individual mismatch factor estimation applied for every intensity.
- [ ] No — *Explain why this information is not reported/not relevant.*

Calculation of spectral mismatch between the reference cell and the devices under test

- [x] Yes — The Enli Technology CO., Ltd. did the calculation of spectral mismatch between the reference cell and the devices.
- [ ] No — *Explain why this information is not reported/not relevant.*

6. Mask/aperture

Size of the mask/aperture used during testing

- [ ] Yes — *Report the size of the mask/aperture.*
- [x] No — We did not use the mask/aperture during testing.

Variation of the measured short-circuit current density with the mask/aperture area

- [ ] Yes — *Report the difference in the short-circuit current density values measured with the mask and aperture area.*
- [x] No — We did not use the mask/aperture during testing for measuring short-circuit current density.

7. Performance certification

Identity of the independent certification laboratory that confirmed the photovoltaic performance

- [ ] Yes — *Identify the independent certification laboratory.*
- [x] No — We didn't do the independent certification. Because the device efficiency achieved in this article is not so high. In addition, our work is mainly focus on the reproducibility of molecular weight issue. The performance certification is not very important in this work.

A copy of any certificate(s)

- [ ] Yes — *Certificate copies should be provided in the Supplementary information. Please state the supplementary item number.*
- [x] No — We didn't do the performance certification via the independent certification laboratory.

8. Statistics

Number of solar cells tested

- [x] Yes — We have tested multiple tens of cells in our lab. The average PCEs were obtained from six independent devices.
- [ ] No — *Explain why this information is not reported/not relevant.*

Statistical analysis of the device performance

- [x] Yes — We give statistical data of the device performance in Fig.3J, Fig. 4C, and Fig. 5a, b, c.
- [ ] No — *Explain why this information is not reported/not relevant.*

9. Long-term stability analysis

Type of analysis, bias conditions and environmental conditions

- [ ] Yes — *Provide a description of the type of analysis, bias conditions and environmental conditions (e.g. illumination type, temperature, atmosphere humidity, encapsulation method, preconditioning temperature, bias) for each long-term stability analysis carried out; see ref. 7 and 8 for details.*
- [x] No — We did not focus on device stability.
